# Invasive Buffelgrass, *Cenchrus ciliaris*, Balances Opportunistic Acquisition of Foliar fungi With Host and Environmental Filtering in Its Introduced Range

**DOI:** 10.1111/mec.17609

**Published:** 2024-12-12

**Authors:** Elizabeth A. Bowman, Christine V. Hawkes, Nathan Jones, Robert M. Plowes, Dino J. Martins, Lawrence E. Gilbert

**Affiliations:** ^1^ Brackenridge Field Laboratory University of Texas at Austin Austin Texas USA; ^2^ Department of Integrative Biology University of Texas at Austin Austin Texas USA; ^3^ Department of Plant and Microbial Biology North Carolina State University Raleigh North Carolina USA; ^4^ Turkana Basin Institute, Stony Brook University Stony Brook NY USA

**Keywords:** *Cenchrus ciliaris*, endophyte, epiphyte, foliar fungi, invasion, native range

## Abstract

Plants host diverse assemblages of fungi on their foliar tissues, both in internal compartments and on exterior surfaces. When plant distributions shift, they can move with their fungal associates (i.e., co‐introduction) or acquire new associates present in the novel environment (host‐jumping). The fungal communities that plants acquire influence a plant's ability to establish and spread in this new environment. Here, we aimed to assess whether invasive *C. ciliaris* hosts similar groups of fungi in its native and introduced ranges and to evaluate community overlap of fungi associated with foliar tissue of *C. ciliaris* and native and non‐native plants within the introduced range. In the introduced range, the majority of OTUs associated with *C. ciliaris* were not found in its native range, although 3.2% of OTUs were common to both ranges. Of these shared OTU, 77.6% were found on co‐occurring natives and non‐natives in the introduced range, whereas 22.4% were unique to *C. ciliaris* indicating a possible co‐introduction. Fungal communities within the introduced range contained a higher proportion of generalist symbionts and increased heterogeneity of foliar communities than in its native range. Within the introduced range, host phylogenetic distance explained more variation than native status. Our findings provide evidence that non‐natives acquire fungi opportunistically from their environment, although host and environmental filtering is present suggesting that successful invasive plants may be able to limit the effect of poor symbionts and select for better ones. Future experimental work will be needed to confirm the occurrence of host selection and identify its mechanisms.

## 1 Introduction

Fungi have influenced terrestrial plant evolution and ecology since plants moved onto land approximately 450 mya (Brundrett 2002; Lutzoni et al. 2018). Associating with above‐ and belowground tissues of all plant lineages studied to date, fungal symbionts can either help or hinder plant establishment. While fungal mutualists can enhance plant tolerance to a range of abiotic and biotic stressors (Arnold and Engelbrecht 2007; Crawford and Hawkes 2020; Karst, Randall, and Gehring 2014) and aid in seedling germination and establishment (Sarmiento et al. 2017), pathogens can decrease host plant fitness impacting their ability to compete for space and proliferate (Colautti et al. 2004; Parker and Gilbert 2007). Therefore, fungi impact the ability of plants to establish and expand into novel environments (Aschehoug et al. 2012), which means they can affect plant range shifts and the success of non‐native species. Historically, the fate of plant introductions has been difficult to predict, and incorporating fungal interactions may improve understanding of these processes.

During range shifts, non‐native plants are introduced into new environments hosting novel fungal communities with which they do not share an evolutionary history. Abrupt and extreme shifts in location can allow colonising plants to escape specialist pathogens—such enemy release is well known (Mitchell and Power 2003). At the same time, non‐native plants in new ranges may lose important mutualists from the home range such as vertically transmitted claviciptaceous endophytes, while acquiring local mutualists that may be either worse or better (e.g., [Reinhart and Callaway 2006]). Following initial introduction, there is a reduction in the fungal endophyte and functional pathogen communities associated with non‐native species; however, with increasing time since the introduction, there is a subsequent increase in the abundance, richness and diversity of these fungal communities (Flory and Clay 2013; Mei et al. 2014). A similar pattern of overall simplification of the bacterial microbiome occupying the foliar tissues was seen in introduced populations of *Centaurea solstitialis* compared to populations in its native range (Lu‐Irving et al. 2019). This evidence from distinct phylogenetic and functional communities of microbes could indicate that a reduction in the complexity of microbial symbiont communities could be a common and widespread pattern post‐introduction. Such simplifications are hypothesised to reduce physiological costs to the plant, thus freeing up resources for growth and reproduction (Lu‐Irving et al. 2019). Whereas a loss of pathogens and herbivores is a well‐studied phenomenon with shifts in plant investment away from defence and into competitive ability (i.e., evolution of increased competitive ability, EICA) (Halbritter et al. 2012; Keane and Crawley 2002; Mitchell and Power 2003), shifts in the entire fungal community can decrease plant investment in maintaining these relationships leading to a similar outcome as seen in EICA. This highlights the importance of considering both harmful and beneficial interactions to avoid missing potential contributions to the success of an invading plant species.

Non‐natives can carry fungal associates from their native range (‘co‐introduction’) or can acquire novel associations with resident fungi (‘host‐jumping’) in the introduced area (Shipunov et al. 2008). Similarity between the invaded and native range in plant community composition and climate may predict how local fungi interact with non‐native plants (Cadotte et al. 2018; Giauque and Hawkes 2016; Gilbert and Parker 2006; Sedio et al. 2020; U'Ren et al. 2012). Host plant identity acts as an important filter for plant‐associated fungi with variation between communities occurring even among intraspecific host genotypes (Bailey et al. 2005; Cordier et al. 2012; Huang et al. 2018; Qian et al. 2018; U'Ren et al. 2019). For example, more closely related plants experience similar levels of disease severity or escape from the same plant pathogens (Gilbert, Briggs, and Magarey 2015). In addition to host identity, foliar fungal communities are strongly shaped by precipitation and temperature (Dea et al. 2022; Giauque and Hawkes 2016; U'Ren et al. 2012), but it is unclear whether plant introductions to similar climates would lead to the acquisition of similar fungal communities as found in their native range. While invasion biology has considered the role microbial communities play in invasion, most work has focused on soil‐ or root‐associated communities of fungi (but see Lu‐Irving et al. 2019).

Understanding how non‐natives acquire foliar fungal communities can assist in predicting long‐term outcomes of invasions on native plant communities. Compared to fungi associating with roots and soil, foliar fungi are likely to be equally or more important in plant invasions given their role in disease resistance and stress tolerance. Based on our knowledge of leaf‐associated fungi in native plants, fungal community assembly tends to have greater potential for both plant‐to‐plant transfer and long‐distance aerial dispersal compared to roots (Lee and Hawkes 2021; Whitaker et al. 2022). Different assembly processes may result in, for example, a different balance of EICA and host‐jumping associations aboveground versus belowground, with consequences for investment in defence versus competitive ability (Blossey and Notzold 1995). These relationships are not necessarily straightforward though. For example, the relative benefits (or negative effects) of local root endophytes for non‐natives and natives is species specific and can switch at different life stages with seed germination and seedling growth impacted more than later growth stages (Geisen et al. 2021). It is challenging to evaluate the role of foliar fungi in invasion given the current scarcity of data on their associations.

In this study, we sought to answer three questions: (1) how an invasive plant's foliar fungal community overlaps between its native and introduced ranges, (2) what drives foliar fungal community composition of *C. ciliaris* within the introduced range compared to co‐occurring native and non‐native plants and (3) whether foliar fungi associated with *C. ciliaris* are co‐introduced or locally assembled. To answer question 1, we surveyed the diversity of foliar fungi associated with the invasive *C. ciliaris* in both its native range of Kenya and its introduced range in Texas and Arizona (Table S1). We expected plants in the two ranges would share the same fungal taxa if those fungi were co‐introduced or were functionally widespread generalists. Otherwise, if local acquisition occurred, we would expect to find a pattern of host‐jumping with *C. ciliaris* associating with novel fungal taxa in the introduced versus native range. To address question 2, we opportunistically sampled leaves of a variety of native and non‐native grasses across Texas that co‐occurred with *C. ciliaris* , which ensured we captured local diversity of both abundant and rare host plants (Table S1). We then analysed to what extent foliar fungal communities were structured by host phylogenetic distance, geographical distance and climate. If the invaded plant community influences the foliar fungal community an invasive plant acquires, we expect host phylogenetic distance to be strongly correlated with fungal community similarity. Additionally, we expected that climate, particularly precipitation, and distance between sampling sites to influence community composition as has been found in other studies of phyllosphere fungi. To address question 3, we evaluated which fungal OTUs were shared between *C. ciliaris* 's native and introduced ranges and what proportion of these OTUs were found on co‐occurring grass hosts within its introduced range. If *C. ciliaris* recruited similar communities to other grass species in the introduced range, then the plant community or abiotic environment in which an invasion occurs plays an important role in structuring the foliar fungi of non‐native plants. Alternatively, if the foliar communities of *C. ciliaris* are distinct from other grass species, then *C. ciliaris* either acquires foliar fungi opportunistically (indicated by highly variable communities and stochastic assembly) or acts as a unique selection filter on the available fungal species pool (indicated by a strong correlation of host phylogenetic distance to community composition or highly repeatable communities).

## 2 Methods

### 2.1 Study Organism

*Cenchrus ciliaris* L. is a perennial C4 grass species native to Africa and western Asia but has been introduced widely as a forage grass. In some regions, it has become an invasive species with detrimental impacts on native biodiversity and ecosystems (Marshall, Lewis, and Ostendorf 2012). Valued as livestock forage and erosion control, *C. ciliaris* is drought tolerant, fire resistant and able to withstand high levels of herbivory (de Albuquerque et al. 2019; Marshall, Lewis, and Ostendorf 2012). It was first introduced in the United States in the early 1900s with a programme for seed production established in Texas in 1945 (Cox et al. 1988; Hanselka 1988). While still widely used for erosion control and as a forage grass, the traits that make *C. ciliaris* economically important have contributed to its invasiveness (Marshall, Lewis, and Ostendorf 2012). Some of the more significant differences observed between *C. ciliaris* in its native African range and locations where it has been introduced are altered fire‐cycles and displacement of native plant communities (Marshall, Lewis, and Ostendorf 2012; McDonald and McPherson 2011).

### 2.2 Sample Collection

**FIGURE 1:**
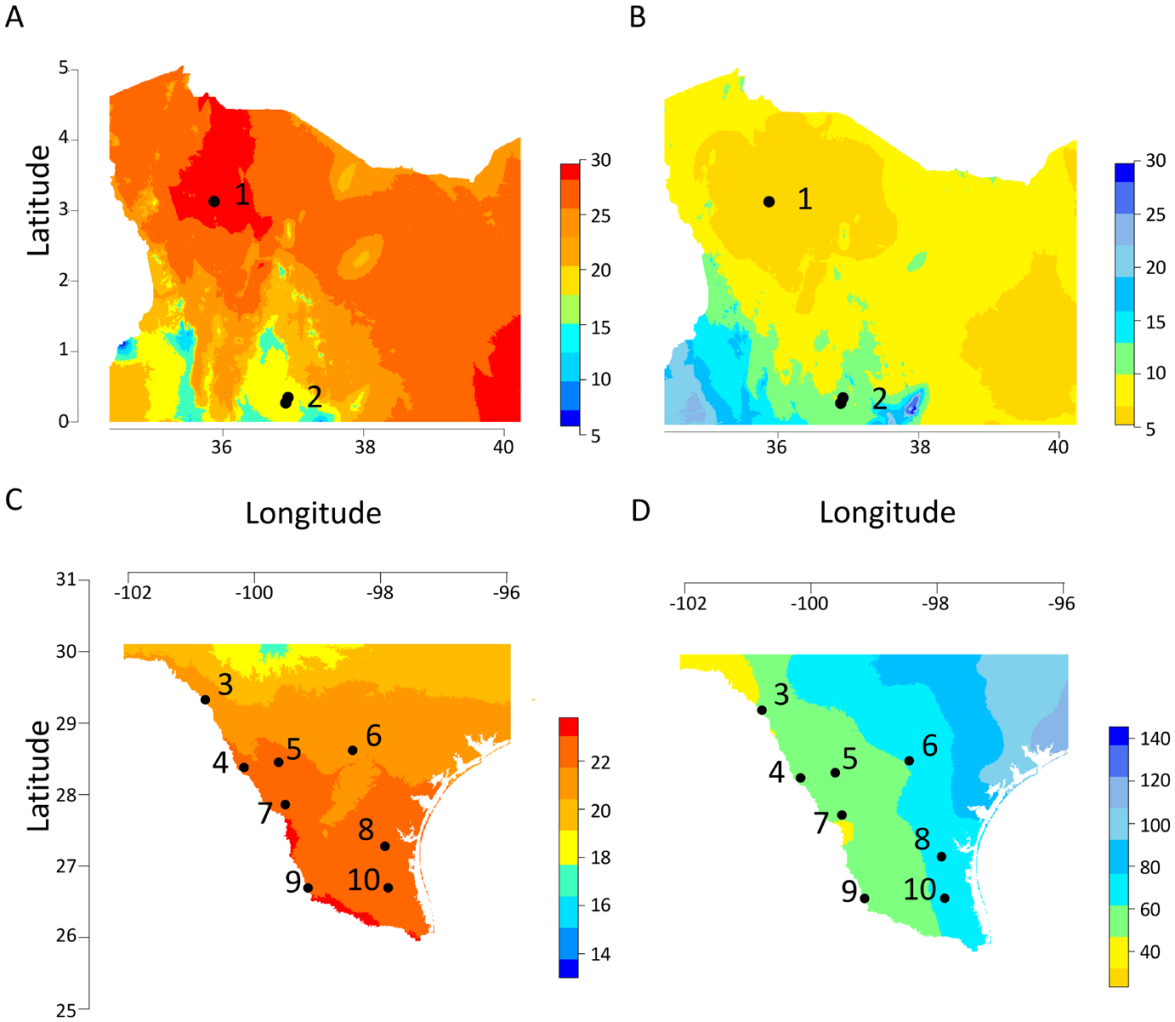
Climatic map showing mean annual temperature (MAT in °C; A, C), mean annual precipitation (MAP in cm; B, D) and sampling sites in Kenya, the native range of *C. ciliaris*, (A, B) and Texas, the introduced range, (C, D). Samples were also collected in Phoenix, Arizona (not shown, Table S1) which has a MAT of 18°C and MAP of 20 cm. (1) Turkana; (2) Mpala; (3) Moody Ranch; (4) Indio Faith; (5) Catarina; (6) Daughtrey; (7) Retama; (8) La Paloma; (9) Falcon Lake; (10) Raymondville. Climate data were downloaded from WorldClim (Fick and Hijmans 2017).

Samples of leaf tissue were collected in 2016 and 2017 from two sites in the native range of *C. ciliaris* in Kenya and nine sites spanning a portion of its introduced range in North America (Figure 1; Table S1). In the introduced range, we also collected leaf tissue opportunistically from eight native and three naturalised grass species (Table S1). For each plant sampled, approximately 10 g of leaf tissue was collected with sterile gloves. The samples were dried at a low temperature before being stored and shipped to Brackenridge Field Laboratory in Austin, TX, USA.

#### 2.2.1 Extraction and Sequencing

Prior to extraction, we cut leaf tissue into 1 cm pieces and homogenised by bead beating. To decrease damage to DNA during bead beating, we froze samples in liquid nitrogen prior to homogenisation. We extracted total genomic DNA from 0.10 g of plant tissue per sample using the DNeasy Plant Kit (Qiagen, USA) and following the manufacturer's instructions. We quantified DNA concentrations on a Qubit (Thermo Scientific, USA) using the Qubit dsDNA High Sensitivity Assay Kit (Thermo Scientific, USA). For all samples, we normalised DNA concentrations to 2 ng/μl and submitted 25 ng of DNA per sample to the University of Texas at Austin Genomics Sequencing and Analysis Facility (GSAF) for amplification and sequencing. Briefly, their procedure was as follows.

Amplification of the region of interest, the inner transcribed spacer region 1 (ITS1), and attachment of Illumina adapters and sequence‐specific barcodes was done through a two‐step process following a standard protocol at the GSAF. In brief, the ITS1 region initially was amplified in triplicate using primers ITS1F (Gardes and Bruns 1993) and ITS2 (White et al. 1990) amended with Illumina adapters (HybITS‐1F_rRNA, 5′‐TCGTCGGCAGCGTCAGATGTGTATAAGAGACAGCTTGGTCATTTAGAGGAAGTAA‐3′ and HybITS2_rRNA, 3′‐CGTAGCTACTTCTTGCGTCGGACAGAGAATATGTGTAGAGGCTCGGGTGCTCTG‐5′). The ITS1 region is not suited to deep phylogenetic analysis due to its highly variable length it nevertheless is well suited to measures of biotic diversity and taxonomic identification (Kõljalg et al. 2013; Nilsson et al. 2018). After amplification, PCR product was pooled before undergoing a second set of PCR reactions in which sequence‐specific barcodes were attached. PCR product was cleaned using AMPure Bead XP purification (Beckman Coulter, USA) based on standard protocols before being pooled and sequenced on Illumina MiSeq v3. All raw sequences were deposited in the NCBI SRA under BioProject ID PRJNA1010801.

#### 2.2.2 Bioinformatics

Post‐sequencing, reads were demultiplexed at the GSAF using standard protocols. We merged forward and reverse reads to create contiguous sequences using USEARCH v.10 (R. C. Edgar 2010) (—fastq_mergepairs; total 3,986,229 merged sequences, 78.62% of read pairs), then assessed sequences for quality using two methods: (1) FastQC (Andrew 2010) to assess the quality of the sequence data and (2) the—fastq‐eestats2 command in USEARCH v.10 using a range of error filters (−ee_cutoffs 0.25, 0.5, 1.0) and length thresholds (−length_cutoffs 150, 300, 10) to assess sequence filtering with these parameters. Using these assessments, we selected a sequence length and maxEE cutoff that would provide high‐quality sequences across samples and maximise read length. We trimmed and filtered reads using—fastq_filter in USEARCH (Edgar 2010) at a maxEE of 0.25 and length of 270 bp resulting in 3,310,850 sequences (83.1% of contigs).

Following quality assessment, we dereplicated sequences using the command ‐fastq_uniques, then checked for chimeras and sequencing errors using the command ‐unoise3 in USEARCH, UCHIME and UNOISE2 (Edgar 2016a, 2016b; Edgar 2010). We clustered sequences into operational taxonomic units (OTUs) with the command‐cluster_smallmem at 95% and 100% sequence similarity resulting in 3465 and 7806 OTUs, respectively (Edgar 2010). We chose two sequence similarity thresholds to confirm that the patterns we were seeing were robust despite not having mock communities to verify our threshold.

We estimated taxonomic placement for sequence data by querying GenBank (Altschul et al. 1990) and UNITE (Kõljalg et al. 2013) with taxonomy parsed with MEGAN5 (Huson et al. 2011). We verified taxonomic placement by phylogenetically placing all sequences with the Tree‐Based Alignment Selector Toolkit (T‐BAS) v2.1 (Carbone et al. 2016). All sequences not within the kingdom Fungi were removed from further analysis (30 OTUs).

To account for differences in sequencing depth between samples, we used coverage‐based rarefaction to normalise sequencing depth without underestimating richness (Chao and Jost 2012; Okazaki et al. 2017). The final dataset had 376 and 597 OTUs clustered at 95% and 100% sequence similarity, respectively. Analyses were conducted with sequences clustered at 95% and 100% sequence similarity. We only present the results for OTUs defined at 95% similarity, because greater stringency did not change our results and 95% similarity is commonly used in studies of foliar fungi (see Results S1 for results based on communities delimited at 100% sequence similarity) (Jumpponen and Jones 2009; U'Ren et al. 2012).

#### 2.2.3 Data Analysis

As we were unable to assess similarity of read abundance to expected biological abundance through inclusion of synthetic or mock communities (Nguyen et al. 2015), we focused our analyses on presence/absence metrics such as OTU richness and the Jaccard index, a dissimilarity measure assessing presence–absence of OTUs between datasets. Unless otherwise specified, all analyses presented here were conducted with OTUs clustered at 95% sequence similarity and with OTUs with < 10 reads removed (U'Ren et al. 2009). All analyses were carried out in R (version 4.2.2) (R Core Team 2021). All analysis data and analysis code are available (see Data Availability Statement below).

#### 2.2.4 Question 1: Comparison of Foliar Fungi Associated With *C. ciliaris* in Its Native and Introduced Ranges

We examined differences in community composition and OTU richness between the ranges and addressed how spatial and abiotic factors acted as drivers of community composition in both ranges. We calculated richness of the foliar fungal community and identified the number of unique and shared OTUs between the ranges. To assess OTU richness associated with *C. ciliaris* in the native (Kenya) and introduced (Texas and Arizona) ranges, we used a linear mixed effects (‘lme’ command in *lme4*, version 1.1.33) model with range as a fixed variable and site as a random variable followed by an analysis of variance (ANOVA) (Bates et al. 2015).

We analysed community composition as a function of range, space and climate with a permutational analysis of variance (PERMANOVA; ‘adonis2’ command in *vegan*, version 2.6.4). To account for spatial autocorrelation, we used a principal coordinate analysis of neighbour matrices (PCNM) for geographical distance (Dray, Legendre, and Peres‐Neto 2006; Legendre and Legendre 2012). Mean annual precipitation (MAP) was used to represent climate, as all climate variables were correlated (Figure S1). The model used was Jaccard distance ~ range * MAP * PCNM; range, MAP and PCNM were fixed effects, and site was added as a random variable with the ‘strata’ command (Dray et al. 2022). We used an ordination via nonmetric multidimensional scaling (NMDS) and a fiddleplot (‘geom_violin’ command in *ggplot2*, version 3.4.0) to visually assess community similarity in the native and introduced range (Wickham 2016). We tested for differences between and within site community dissimilarity using a Kruskal–Wallis test. To assess higher level taxonomic fidelity (i.e., is *C. ciliaris* associated with the same classes of fungi in both ranges), we evaluated differences between the introduced and native range at the class level with a Chi‐square test. For this analysis, we used counts of OTUs belonging to each class.

#### 2.2.5 Question 2: Comparison With Foliar Fungal Communities Associated With Co‐Occurring Grass Species in the Introduced Range

To compare foliar fungal communities of *C. ciliaris* and other plant species in the introduced range, we limited our analyses to samples collected in Texas, USA (Table S1). Here, we conducted analyses to determine what structures the foliar fungal community of *C. ciliaris* compared with co‐occurring native and non‐native grasses.

We conducted a PERMANOVA with Jaccard dissimilarity of the foliar fungal community to assess contribution of host phylogenetic distance, geographical distance and climate to fungal community variation. Site was added in as a random variable as above. We were unable to include the native status of host plants (i.e., native vs. non‐native) within the model as it was correlated to phylogenetic distance (Kruskal–Wallis test: *χ* ^2^ = 32.8, *p* < 0.0001). Geographical distance was calculated as above. Climate was represented by MAP, as above. To calculate host plant phylogenetic distance, we used the region encompassing the *matK* (maturease K) coding gene and the *trnK* (tRNA‐Lys) introns which were downloaded from GenBank (Table S2) (Aliscioni et al. 2012; Coordinators 2018). Sequences were concatenated, aligned in MAFFT version 7 (Katoh, Rozewicki, and Yamada 2018) using a tree‐based progressive model (FFT‐NS‐2) and trimmed in Mesquite version 3.6 (Maddison and Maddison 2023). Using this sequence data, we created a phylogeny in MegaX version 10.2.4 (Kumar et al. 2018), calculated the phylogenetic distance based on the phylogenetic tree (‘cophenetic.phylo’ in *ape* version 5.7.1) (Paradis and Schliep 2019) and used a principal coordinates analysis to transform the pairwise patristic distance matric to phylogenetic vectors (‘cmdscale’ command in *stats* version 4.3.0) (R Core Team 2021). Data were visualised using an NMDS with Jaccard's distance as described earlier showing the fungal community associated with all samples collected within the introduced range.

#### 2.2.6 Question 3: Evaluation of How *C. ciliaris* Acquires Fungal Symbionts in Its Introduced Range

Here, we assessed to what extent fungal OTUs associated with *C. ciliaris* in both its native and introduced range were possible co‐introductions or represented host‐jumping events. We used three approaches to address these two options.

First, we examined indicator species (i.e., fungal OTU) associated with *C. ciliaris within* each range, the strength of these associations and to what extent trophic mode may have changed across communities in the two ranges (i.e., was there a loss of pathogens and mutualists in the introduced range and an increase in generalists). We would expect that there would be a decrease in the number of indicator species in the introduced range relative to the native range if host‐jumping occurred due to an increase in opportunistic associations. We ran an indicator species analysis (‘multipatt’ command in *indicspecies*, R package version 1.7.12) on presence/absence data with the association index ‘r.g’ (De Cáceres and Legendre 2009; De Cáceres, Legendre, and Moretti 2010) to assess which OTUs were more often found in each range. The association index ‘r.g.’ not only takes into account what OTUs are maximally associated with each range, but also considers combinations of the groups (i.e., OTUs associated with both ranges) which can inform whether there are any OTU that overlap the two ranges (e.g., possible co‐introductions) (De Cáceres, Legendre, and Moretti 2010). To assess differences in the putative function of OTUs, we defined functional guilds for the OTUs at the genus level using fun^fun^ (Flores‐Moreno et al. 2019).

To measure how community stability changed between ranges, we compared the OTU co‐occurrence patterns in each range. Decreased co‐occurrences in the introduced range indicate possible opportunistic acquisition of fungal associates (i.e., host‐jumps), whereas increased co‐occurrences (positive or negative) could indicate co‐introductions, as well as community assembly processes or facilitation. We used a probabilistic model with the R package *cooccur* version 1.3 (Griffith, Veech, and Marsh 2016; Veech 2013). The model allows the comparison of observed co‐occurrence and expected co‐occurrence of OTUs in order to assess whether the observed frequency of co‐occurrence is equal to expected co‐occurrence (i.e., random association), greater than expected (positive association) or less than expected (negative association) based on an alpha of 0.05 (Griffith, Veech, and Marsh 2016). We conducted separate analyses for the native and introduced range to determine how associations with the foliar community might differ between the two ranges. There were 758 species pairs and 2336 pairs analysed in the native and introduced ranges, respectively. To account for a deeper sampling depth in the introduced range (Table S1), we confirmed our results by re‐running the analyses for the introduced range 100 times after randomly subsampling as equal number of subsamples as the native range (*n* = 27).

Finally, we compared OTUs associated with *C. ciliaris* and shared in both ranges to OTUs found on other grass species within the introduced range to determine whether the shared OTUs represented cases of co‐introduction or were generalist, cosmopolitan species. To determine whether *C. ciliaris* acquired more heterogeneous communities (i.e., opportunistic host‐jumping associations) compared to co‐occurring plants, we compared dissimilarity of fungal communities at the OTU and class level across native status. We categorised samples by whether the host plant was native or introduced (non‐native) keeping all plant species. Using the Kruskal–Wallis test, we assessed dissimilarity of foliar fungal communities between native and non‐native grasses, as well as within each group based on native status. We analysed the composition of communities at the class level with a Chi‐square test with counts of OTUs assigned to each class, as above. Finally, to determine whether the 76 fungal OTUs shared between *C. ciliaris* 's native and introduced range were potentially cosmopolitan species (and putative co‐introductions), we compared these OTUs with OTUs associated with other native and non‐natives within the introduced range.

## 3 Results

### 3.1 Question 1: Comparison of Foliar Fungi Associated With *C. ciliaris* in Its Native and Introduced Ranges

We documented 236 OTUs species of fungi associated with leaf tissues of *C. ciliaris* (91 OTUs with greater than 10 occurrences). The individuals in the introduced range harboured slightly higher species richness (16 ± 7 OTU) than those in the native range (14 ± 6 OTU), although the difference was not significant (*F* = 0.1369). Including singletons, we found 76 OTUs (32.2%) shared between *C. ciliaris* 's native range in Kenya and its introduced range in the United States, whereas 108 (45.7%) were unique to *C. ciliaris* in the introduced range and 52 (22.0%) in the native range.

**FIGURE 2:**
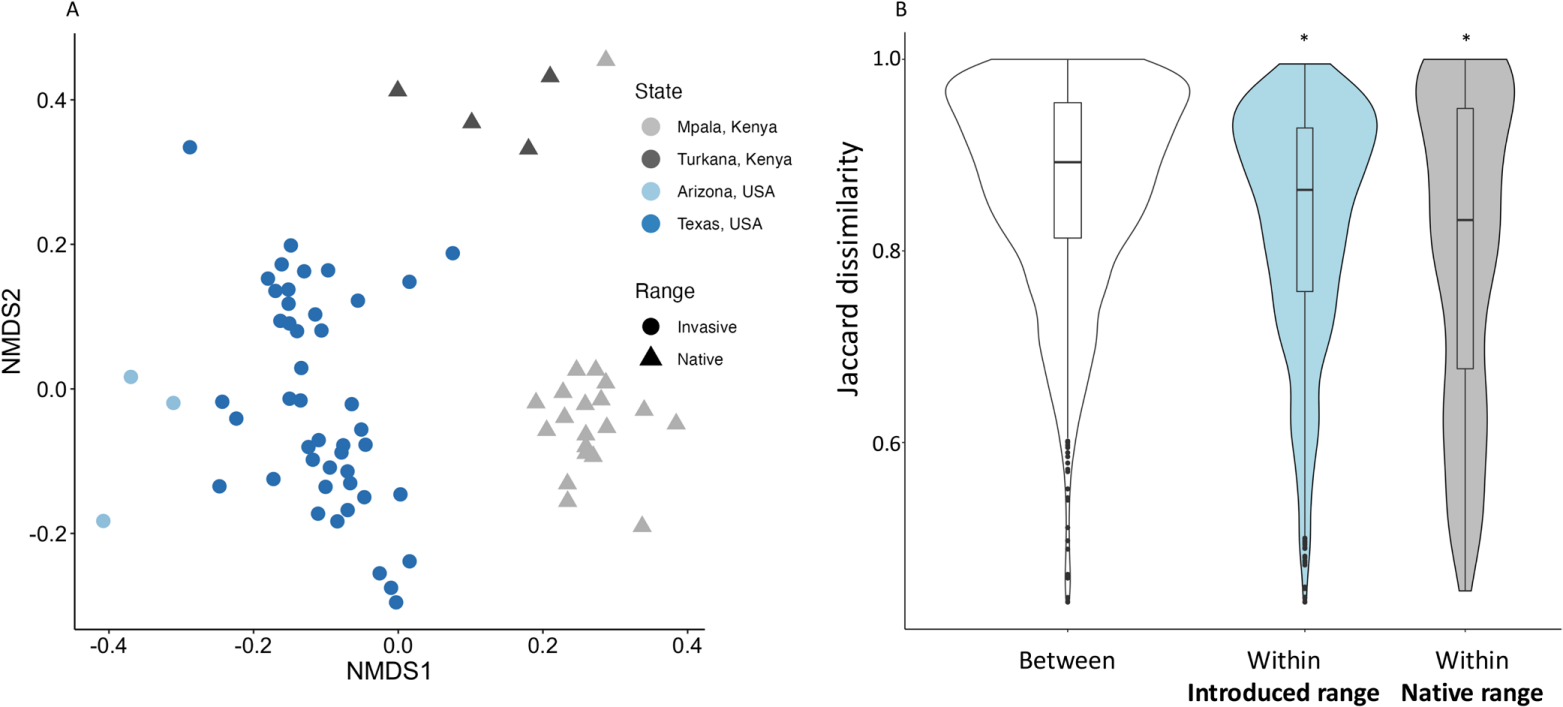
Comparison of foliar fungal communities associated with *C. ciliaris* in its native range, Kenya and its introduced range, Texas and Arizona. (A) is an NMDS ordination showing community similarity across both ranges (stress = 0.2480). (B) is a pairwise comparison of fungal communities associated with *C. ciliaris* across the native and introduced range (Overall) and within each range (Introduced and Native range). The astericks signify a significant different from "Between groups" as determined with a Kruskal‐Wallis test.

**FIGURE 3:**
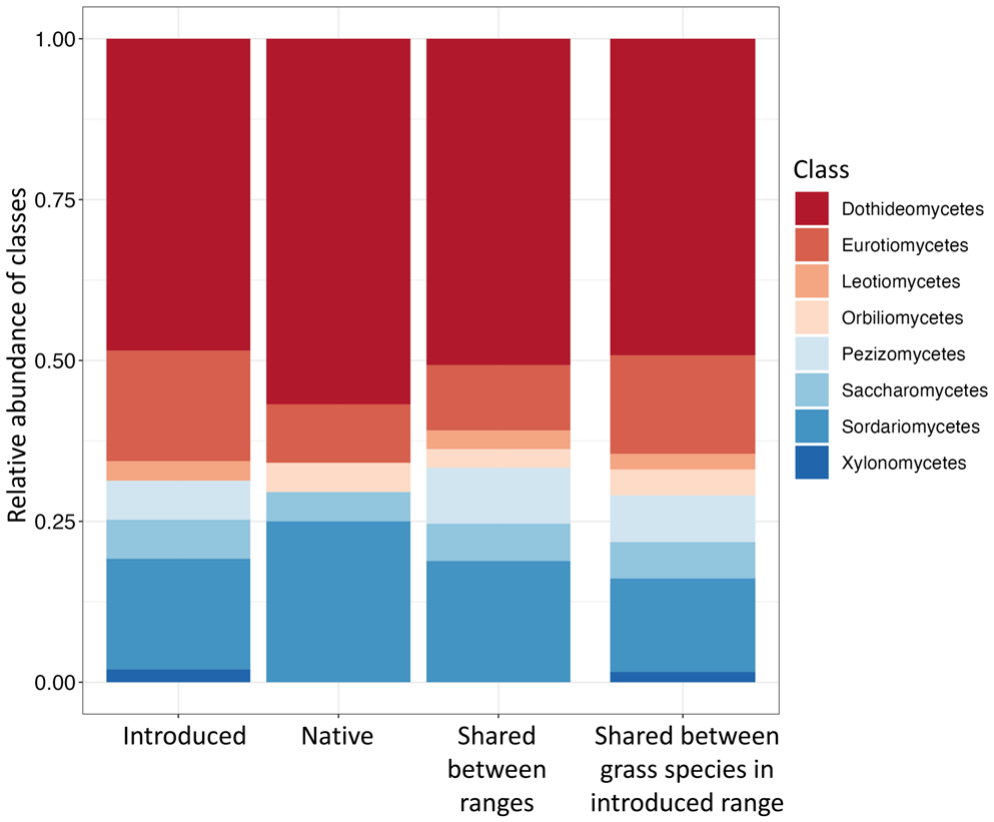
Taxonomic classification of OTUs at the class level. ‘Introduced’ and ‘Native’ categories display OTU associated with *C. ciliaris* that are unique to each range. ‘Shared between ranges’ indicates OTU associated with *C. ciliaris* and shared between the two ranges. ‘Shared between grass species in introduced range’ displays OTU shared across all grass species within the introduced range.

Overall, range explained 8.3% of the variation in fungal community composition associated with *C. ciliaris* (*p* = 0.0430) (Figure 2A; Table 1). Consistent with this, foliar fungal communities were more similar within each range (Jaccard index, native range: 0.79 ± 0.2; introduced range: 0.82 ± 0.1; Kruskal–Wallis test: ${Χ}_{1}^{2}$ = 1.4, *p* = 0.2306) than between the native and introduced range (0.87 ± 0.1; Kruskal–Wallis test: ${Χ}_{1}^{2}$ = 186.7, *p* < 0.0001) (Figure 2B). MAP explained 4.5% of the variation in communities of foliar fungi (Table 1). When we considered fungal communities at the class level, the same classes of fungi where present in both ranges (Χ_10_ = 10.9, *p* = 0.3580) (Figure 3). In both ranges, *C. ciliaris* hosted foliar fungi predominantly from the class Dothideomycetes, Eurotiomycetes and Sordariomycetes. There were no OTUs belonging to Pezizomycetes unique to the native range.

**TABLE 1:** PERMANOVA analysis of variation in the foliar community associated with *C. ciliaris* in its introduced range and native range.

	*F*‐statistic	*p*	*R* ^2^
Range	6.98	0.043	0.083
Geographical distance	1.58	0.134	—
MAP	3.77	0.037	0.045
Range*Geo. dist.	1.75	0.069	—
Range*MAP	3.32	0.516	—
Geo. dist.*MAP	1.99	0.115	—
Range*Geo. dist.*MAP	1.25	0.115	—
Total variation explained			0.129

*Note:* The model used here was range*geographical distance (geo. dist.)*mean annual precipitation (MAP) with site as a random variable.

### 3.2 Question 2: Comparison With Foliar Fungal Communities Associated With Co‐Occurring Grass Species in the Introduced Range

**FIGURE 4:**
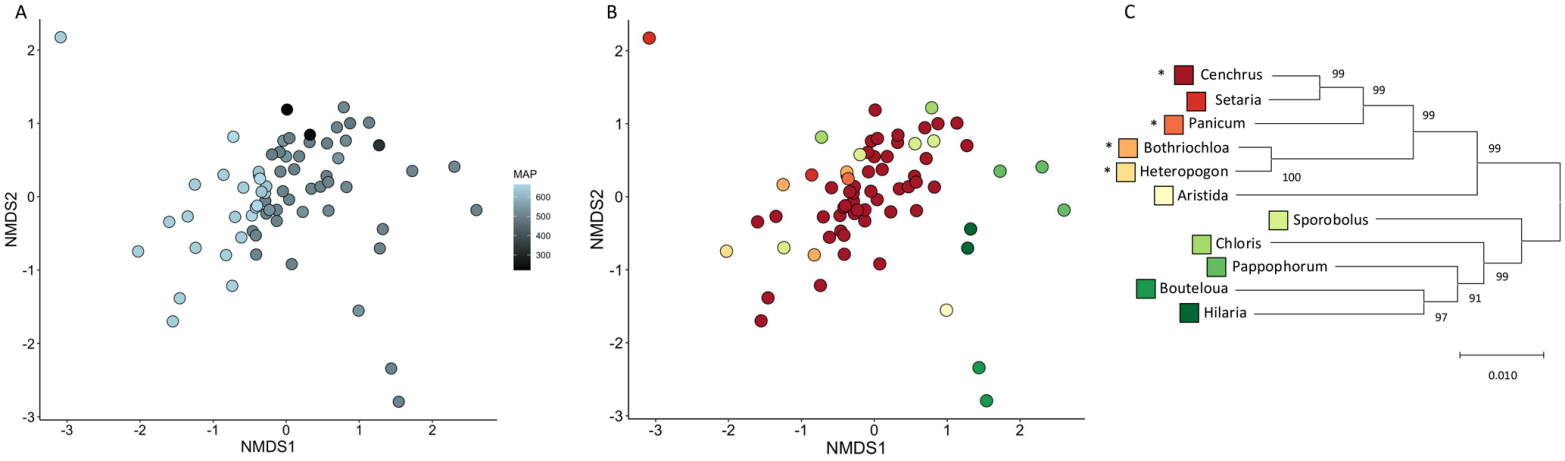
NMDS ordinations of foliar fungal communities associated with grass species sampled across Texas (stress 0.1604). (A) shows points coloured based on mean annual precipitation (MAP). (B) shows points coloured by host species represented in the phylogeny in (C). Numbers at branch points are bootstrap support values. *non‐native.

Within the introduced range, we isolated 225 OTUs at 95% sequence similarity (109 OTUs with greater than 10 occurrences). Total variation in fungal community composition explained by host phylogenetic distance, climate, and geographical distance was 19.6%. MAP explained the most variation in communities of foliar fungi associated with both native and invasive grass species (7.4%) followed by host phylogeny (6.5%) (Figure 4; Table 2).

**TABLE 2:** PERMANOVA analysis of variation in the foliar community associated with plant species in the introduced range.

	*F*‐statistic	*p*	*R* ^2^
Host phylogeny	5.16	0.001	0.065
MAP	5.86	0.007	0.074
Geo. dist.	1.72	0.005	0.022
Host phylogeny*MAP	1.30	0.043	0.016
Host phylogeny*geo. dist.	1.14	0.427	—
MAP*geo. dist.	1.50	0.003	0.019
Host phylogeny*MAP*geo. dist.	1.35	0.343	—
Total variation explained			0.196

*Note:* The model used here was host phylogeny*mean annual precipitation (MAP)*geographical distance (geo. dist.) with site as a random variable.

### 3.3 Question 3: Evaluation of How *C. ciliaris* Acquires Fungal Symbionts in Its Introduced Range

There were 22 OTUs (42.3% of native range OTU) that were indicator species of the native range and 21 OTUs (19.4% of introduced range OTU) were indicators of the introduced range of *C. ciliaris* (Table 3). The majority of indicator OTUs in both ranges were identified as Dothideomycetes (59% native range, 68% introduced range) in the orders Pleosporales or Capnodiales. Only a small proportion (22.9%) of OTUs were found in the fun^fun^ database, even when we classified OTUs at the genus level, limiting our ability to draw inferences (Table S3). Compared to the native range, the introduced range had a higher proportion of pathotrophic and saprotophic OTUs and showed a loss of symbiotrophic fungi (Figure S2). OTUs shared between ranges by *C. ciliaris* showed similar trophic modes as the native range (Figure S2).

**TABLE 3:** OTUs defined as indicator species within the native and invasive range.

Range	Otu	IndVal	*p*	*A*	*B*	Class	Order	Family	Genus
Native	Otu1345	0.79	0.0010	1.00	0.63	Dothideomycetes	Capnodiales	Cladosporiaceae	Cladosporium
Introduced	Otu1385	0.73	0.0020	0.88	0.60	Dothideomycetes	Pleosporales	Pleosporaceae	Exserohilum
Introduced	Otu2720	0.72	0.0010	1.00	0.52	Eurotiomycetes	Chaetothyriales	Herpotrichiellaceae	Minimelanolocus, Exophiala
Native	Otu2723	0.71	0.0010	1.00	0.50	Dothideomycetes	Pleosporales	Didymosphaeriaceae	Pseudocamarosporium
Introduced	Otu105	0.67	0.0030	0.89	0.50	Dothideomycetes	Pleosporales	Pleosporaceae	Curvularia
Introduced	Otu792	0.60	0.0030	0.95	0.38	Taphrinomycetes	Taphrinales	Taphrinaceae, Protomycetaceae	Taphrina, Protomyces
Native	Otu665	0.58	0.0010	0.90	0.38	Dothideomycetes	Pleosporales	Pleosporaceae	Curvularia
Introduced	Otu336	0.58	0.0020	0.94	0.35	Dothideomycetes	Capnodiales	Cladosporiaceae, Davidiellaceae	Cladosporium, unclassified
Native	Otu333	0.58	0.0010	1.00	0.33	Pezizomycetes	Pezizales	Ascobolaceae	Ascobolus, Saccobolus
Introduced	Otu206	0.56	0.0070	0.94	0.33	Dothideomycetes	Pleosporales	Didymosphaeriaceae	Pseudocamarosporium
Introduced	Otu348	0.54	0.0250	0.89	0.33	Dothideomycetes	Capnodiales	Neodevriesiaceae	Neodevriesia
Introduced	Otu179	0.54	0.0050	0.94	0.31	Dothideomycetes	Dothideales	Aureobasidiaceae	Aureobasidium
Introduced	Otu20	0.54	0.0110	0.94	0.31	Dothideomycetes	Pleosporales	Lentitheciaceae	Murilentithecium
Introduced	Otu34	0.54	0.0070	1.00	0.29	Dothideomycetes	Pleosporales	Trematosphaeriaceae	Medicopsis
Introduced	Otu222	0.52	0.0190	0.93	0.29	Dothideomycetes	Pleosporales	Phaeosphaeriaceae	
Introduced	Otu472	0.50	0.0060	1.00	0.25	Dothideomycetes	Capnodiales	Teratosphaeriaceae	Acrodontium
Introduced	Otu796	0.50	0.0080	1.00	0.25	Taphrinomycetes	Taphrinales	Taphrinaceae, Protomycetaceae	Taphrina, Protomyces
Native	Otu1392	0.49	0.0030	0.73	0.33	Taphrinomycetes	Taphrinales	Taphrinaceae, Protomycetaceae	Taphrina, Protomyces
Introduced	Otu33	0.48	0.0140	1.00	0.23	Dothideomycetes	Pleosporales	Phaeosphaeriaceae	Phaeosphaeria
Introduced	Otu1379	0.48	0.0180	1.00	0.23	Dothideomycetes	Pleosporales	Pleosporaceae	Curvularia
Introduced	Otu773	0.48	0.0120	1.00	0.23	Pezizomycetes	Pezizales	Pezizaceae, Ascobolaceae, Pyronemataceae	Ascobolus, Peziza, Marcelleina, Cazia, Saccobolus, Iodophanus, Pachyella
Introduced	Otu341	0.46	0.0150	1.00	0.21	Sordariomycetes	Hypocreales	Stachybotryaceae	Stachybotrys
Native	Otu178	0.46	0.0050	1.00	0.21	Dothideomycetes	Pleosporales	Pleosporaceae	Curvularia
Native	Otu783	0.45	0.0280	0.70	0.29	Dothideomycetes	Pleosporales	Phaeosphaeriaceae	Parastagonospora
Introduced	Otu1330	0.43	0.0310	1.00	0.19	Dothideomycetes	Capnodiales	Cladosporiaceae, Davidiellaceae	Cladosporium, unclassified
Introduced	Otu476	0.43	0.0310	1.00	0.19	Dothideomycetes	Pleosporales	Phaeosphaeriaceae, unclassified, Leptosphaeriaceae	
Introduced	Otu800	0.43	0.0340	1.00	0.19	Pezizomycetes	Pezizales	Pezizaceae, Ascobolaceae, Pyronemataceae	Ascobolus, Peziza, Marcelleina, Cazia, Saccobolus, Iodophanus, Pachyella
Native	Otu791	0.42	0.0160	0.83	0.21	Dothideomycetes	Pleosporales	Pleosporaceae	Curvularia
Native	Otu1324	0.42	0.0120	0.83	0.21	Dothideomycetes	Capnodiales	Cladosporiaceae, Davidiellaceae	Cladosporium, unclassified
Introduced	Otu2772	0.41	0.0470	1.00	0.17	Dothideomycetes	Pleosporales		
Native	Otu2733	0.41	0.0080	1.00	0.17	Dothideomycetes	Pleosporales	Phaeosphaeriaceae	Septoriella
Native	Otu352	0.41	0.0090	1.00	0.17	Dothideomycetes	Pleosporales	Phaeosphaeriaceae	Phaeosphaeria
Native	Otu248	0.41	0.0110	1.00	0.17	Eurotiomycetes	Chaetothyriales	Cyphellophoraceae	Cyphellophora
Native	Otu2721	0.41	0.0060	1.00	0.17	Pezizomycetes	Pezizales	Pezizaceae, Ascobolaceae, Pyronemataceae	Ascobolus, Peziza, Marcelleina, Cazia, Saccobolus, Iodophanus, Pachyella
Native	Otu271	0.39	0.0360	0.71	0.21	Cladosporium	Cladosporiaceae	Capnodiales	Dothideomycetes
Native	Otu662	0.37	0.0430	0.80	0.17	Dothideomycetes	Capnodiales	Cladosporiaceae	Cladosporium
Native	Otu40	0.37	0.0360	0.80	0.17	Dothideomycetes	Pleosporales	Phaeosphaeriaceae	Septoriella
Native	Otu219	0.35	0.0400	1.00	0.13	Dothideomycetes	Pleosporales	Phaeosphaeriaceae	Phaeosphaeria
Native	Otu1250	0.35	0.0320	1.00	0.13	Dothideomycetes	Capnodiales, unclassified	Capnodiaceae, Mycosphaerellaceae, unclassified, Teratosphaeriaceae	
Native	Otu1264	0.35	0.0340	1.00	0.13	Pezizomycetes	Pezizales	Pezizaceae, Ascobolaceae, Pyronemataceae	Ascobolus, Peziza, Marcelleina, Cazia, Saccobolus, Iodophanus, Pachyella
Native	Otu2248	0.35	0.0330	1.00	0.13	Sordariomycetes	Hypocreales	Stachybotryaceae	Alfaria
Native	Otu2727	0.35	0.0360	1.00	0.13	Sordariomycetes	Hypocreales	Unclassified	Acremonium
Native	Otu2734	0.35	0.0300	1.00	0.13	Sordariomycetes	Hypocreales	Unclassified	Acremonium

*Note:* Here, all OTU with a significant *p*‐value were included despite low IndVal scores for transparency.

**FIGURE 5:**
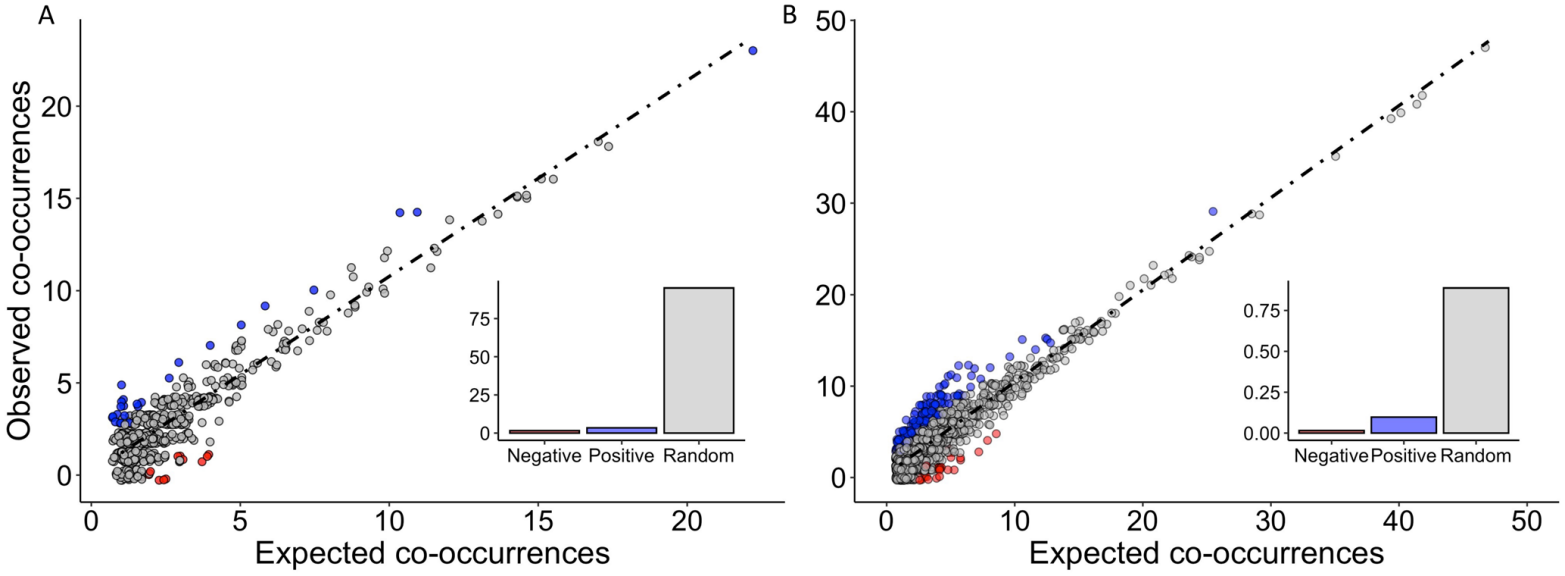
Co‐occurrence analysis of OTU associated with foliar tissues of *C. ciliaris* in the native (A) and introduced (B) range. Grey represents random associations in which expected co‐occurrences of species pairs was equal to observed co‐occurrences; blue represents positive associations in which observed co‐occurrences was greater than expected; red represents negative associations in which observed co‐occurrences was less than expected.

Co‐occurrence analyses indicated that in both the native and introduced ranges, most species pairs occurred randomly. In the native range, 5.0% of species pairs were non‐random with 3.4% of the non‐random pairs observed more often than expected (i.e., positive associations) and only 1.6% observed less often than expected (i.e., negative associations) (Figure 5A). In contrast, 11.3% of species pairs were non‐random in the introduced range with a majority of the non‐random pairs qualifying as positive associations (9.8%) and an equal number as in the native range (1.6%) as negative associations (Figure 5B). When we subsampled the introduced range to an equal sampling depth as in the native range, we found similar results as when the whole dataset was used with 6.4% ± 1.1% of species‐pairs occurring more often than expected and 0.8% ± 0.4% less than expected (Table S4).

**FIGURE 6:**
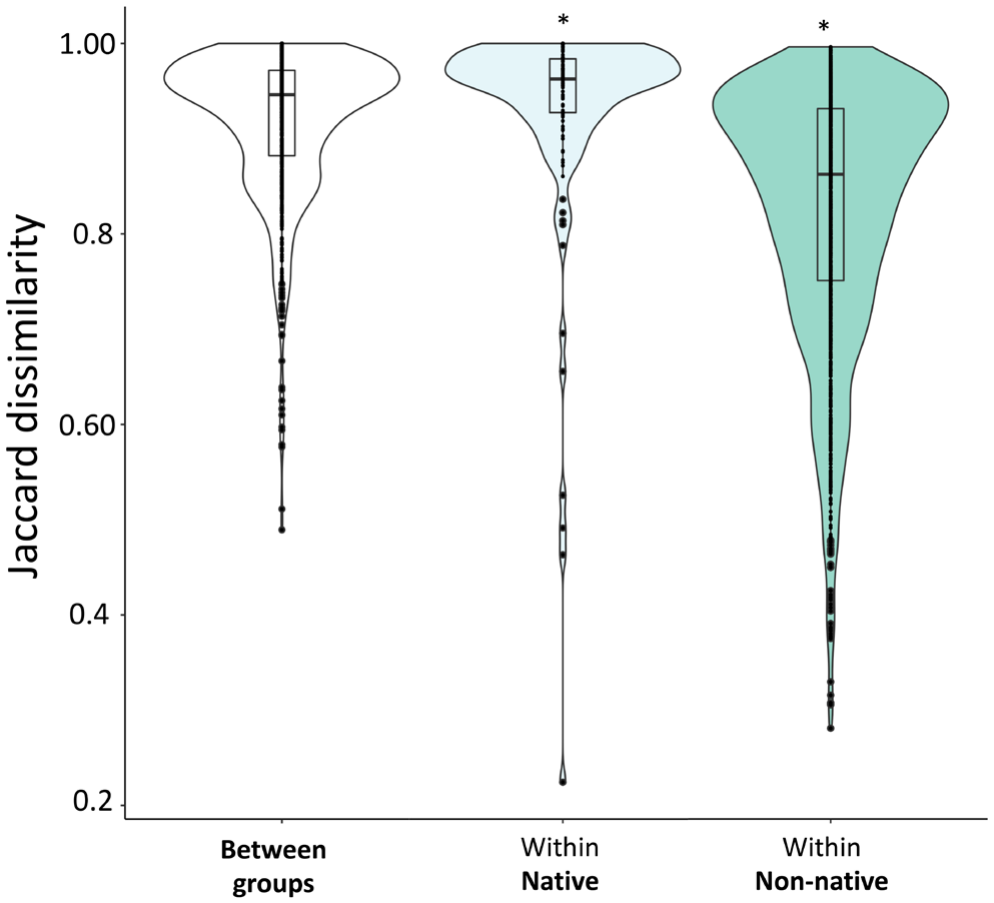
Pairwise comparison of fungal communities associated with plant species native to Texas and non‐native species native to Africa. See Table S1 for list of plant species. The astericks signify a significant difference from "Between groups" as determined with a Kruskal‐Wallis test.

Within the Texas‐introduced range, fungal communities associated with non‐native species (Jaccard dissimilarity: 0.82 ± 0.1) were significantly more similar than communities associated with just native species (Jaccard dissimilarity: 0.93 ± 0.1; Kruskal–Wallis: $\chi_{1}^{2}$ = 260.6, *p* < 0.0001; Figure 6) and between native and non‐native groups (Jaccard dissimilarity: 0.92 ± 0.1; Kruskal–Wallis: $\chi_{1}^{2}$ = 586.5, *p* < 0.0001; Figure 6). *C. ciliaris* shared more OTUs with plant species native to Texas (63.5%) than non‐native species (38.6%). The most common shared classes of OTUs between all grasses in the introduced range were Dothideomycetes, Sordariomycetes and Eurotiomycetes (Figure 3). Composition of foliar fungal communities at the class level did not differ between *C. ciliaris* and native and non‐native grasses (Χ_14_ = 9.2, *p* = 0.8148). Of the 76 foliar fungal OTUs associated with *C. ciliaris* in its native and introduced range, 59 were found on other native and non‐native species within the United States (Figure S3). The 17 OTUs truly unique to *C. ciliaris* were found to be low in relative abundance (Table S5).

## 4 Discussion

Our study indicates that *Cenchrus ciliaris* , a successful invader across tropical and subtropical arid ecosystems, not only acquires a majority of its foliar fungal associates opportunistically in its introduced range, but also is co‐introduced with a small number of fungal OTUs that seem to be in low abundance. Fungal communities within the introduced range contained a higher proportion of generalist symbionts and an increase in heterogeneity of foliar communities than in its native range (Figures 2B and 3). In general, the non‐native grasses we examined showed lower host specificity than seen in co‐occurring natives (Figure 6). An opportunistic acquisition of fungal associates and the breakdown of the more structured symbiotic relationships likely are due to the lack of a shared evolutionary history during introductions. A shared evolutionary history increases the rewards of mutualistic interactions (Batstone et al. 2020; Gehring et al. 2017) and reduces the costs of antagonistic interactions (Gilbert and Parker 2010). With this, one would expect then that movement to a novel area would decrease the fitness of non‐native plant species and limit their ability to establish and spread, but here we see the opposite with *C. ciliaris* a highly successful invasive plant. Although a number of fungi found on *C. ciliaris* leaves in its introduced range are purported pathogens, at the time of collection the samples showed no signs or symptoms of disease. This could indicate that, although classified as pathogens, *C. ciliaris* could be a non‐susceptible host. The parallel loss of pathogens and symbiotrophs, as well as the observed high fitness level (i.e., invasiveness) could support a shift of carbon investment from support of symbiotic associations towards growth and reproduction similar to what has been seen with EICA and what would be expected under the endophyte‐enemy release (EER) hypothesis in which the absence of co‐evolved mutualists similarly frees up carbon for growth, reproduction and competition (Evans 2008).

Because host‐jumping dominates the fungal associations of *C. ciliaris* in its introduced range, it is likely that this grass has lost mutualists, which is reinforced by an increase in within‐species community dissimilarity in its introduced range (Figure 2B) and an increase in positive co‐occurrences (Figure 5). A loss of co‐evolved symbionts can be beneficial if there is (1) a reduction in carbon costs that come from mutualisms without a loss of fitness (Wyatt et al. 2014), (2) a loss of natural enemies such as pathogens and herbivores (Enemy Release Hypothesis; Colautti et al. 2004) and/or (3) concurrent acquisition of novel mutualists that confer improved fitness in the introduced range (Reinhart and Callaway 2006). If the introduced environment is similar to the native range and there is a decrease in negative impacts from pathogens and herbivores, non‐natives could establish and spread without their mutualists. Kenya and the Southwestern United States both host savanna plant communities dominated by similar families (Poaceae and Fabaceae) and are dry, hot climates with seasonal precipitation which could contribute to the selection of similar groups of fungi to be present (Huang et al. 2018).

When foliar fungal communities were compared across native and non‐native species in the introduced range, natives had more heterogeneous communities than non‐natives (Figure 6). These differences likely reflect stronger native filtering of foliar fungi at the species level and further support the idea that non‐natives acquire opportunistic associates. Although we observed a multiyear smut outbreak in the native range of *C. ciliaris* , we have not identified any significant disease in the introduced ranges in South Texas (Rhodes et al. 2024). The low incidence of disease in *C. ciliaris* in its introduced range despite invading grasslands dominated by confamilial species provides support for a symbiotic mismatch (but see Makiela and Harrower 2008; Perrott and Chakraborty 1999; Rodriguez et al. 1999). A similar reduction in herbivory from arthropods has been observed on *C. ciliaris* in its introduced range compared to its native range (Morrison et al. 2023). In general, disease impact shows a strong signature of co‐evolution with phylogenetic relatedness of plant communities influencing disease pressure within the communities (Parker et al. 2015). This could indicate that although pathogens are able to colonise internal and external leaf compartments, non‐natives are tolerant of this infection which results in a limited physiological impact (Bénard, Vavre, and Kremer 2020).

The consistent dominance of fungal communities by members of Dothideomycetes across all sampled grasses could be due to a long shared co‐evolutionary history or a predominance of this class in these ecosystems due to abiotic and biotic filtering. Leaf communities associated with other native and non‐native grasses in the introduced range were similarly dominated by Dothideomycetes (Figure 3), as were the co‐introduced OTUs (Table S5). Plants have a long evolutionary history closely tied to fungal evolution with the diversification of the subphylum Pezizomycotina coinciding with that of embryophytes (Lutzoni et al. 2018). A study of endophytic fungi in Arizona showed that fungi belonging to the class Dothideomycetes are dominant members of endophytic fungal communities of angiosperms and are more common in low elevation areas (< 750 m above sea level) (Huang et al. 2018). Dothideomyceteous fungi could dominate sampled areas in both the native and introduced range as these areas are low in elevation and predominately covered by grasslands. Within the Dothideomycete class, there was coarse fidelity at the order level with Pleosporales and Capnodiales the most common—35.5% ± 13.6% and 24.3% ± 11.1%, respectively. A survey of foliar endophyte communities in *Panicum hallii* , a widespread C_4_ perennial grass in southwestern United States and Mexico, was similarly dominated by Dothideomycetes with Pleosporales one of the most abundant orders (Giauque and Hawkes 2016). Here, we find evidence that invasive *C. ciliaris* hosts a similar community due to environmental and ecological filtering, but the ability of *C. ciliaris* to successfully spread in the introduced range might suggest that any ‘cost’ of hosting foliar fungi may be reduced. We did not directly measure performance here, and explicit tests of plant–fungal interactions would be needed to confirm this mechanism.

Although our experimental design allowed for a robust assessment of invasion processes with regard to fungi, we recognise that we are sampling established non‐natives and missed the initial invasion, where patterns could have been different. For instance, a higher proportion of the fungal community could have been co‐introduced with *C. ciliaris* that has since been diminished. Nevertheless, by sampling a grass that was introduced approximately 100 years ago, we are able to assess long‐term patterns. We found that despite this relatively long time since introduction, *C. ciliaris* has not yet acquired a unique foliar fungal community and, observationally, has not shown symptoms of disease (Flory and Clay 2013; Hawkes 2007). This is surprising as it is invading a community dominated by diverse C4 grass species indicating that there is either enough of a phylogenetic distance between *C. ciliaris* and co‐occurring grass species, that *C. ciliaris* possesses a novel physical or chemical defence, or the community it does acquire is composed of generalist fungi.

Our study sheds light on the dynamic interactions between the invasive plant species *C. ciliaris* and its foliar fungal associates in both its native and introduced ranges. Our findings indicate that foliar fungal communities are locally assembled in *C. ciliaris'* introduced range through environmental and ecological filtering. Only a small proportion of OTUs were possibly co‐introduced, and they occur at low abundances (Table S5). The high fitness of *C. ciliaris* in its introduced range accompanied with a lack of observed pathogens and evidence for their seeming opportunistic acquirement of foliar fungi suggests that the loss of carbon cost from antagonistic and mutualistic interactions (i.e., the EICA and EER hypothesis) could be driving the invasion success of *C. ciliaris* , but more pointed research should be done to investigate this further. Understanding the mechanisms behind the assembly of foliar fungal communities in non‐native grasses can provide insights into community assembly processes, the development of host‐specific associations such as pathogens and mutualists and the role they play in invasion outcomes.

## Author Contributions

E.A.B. processed raw data, conducted the analyses and wrote the manuscript. N.J., C.V.H., R.M.P., D.J.M. and L.E.G. designed the experiment, collected samples and edited the manuscript. NJ assisted in conducting analyses.

## Conflicts of Interest

The authors declare no conflicts of interest.

## Supporting information


Appendix S1.

## Data Availability

All metadata, processed data and code for the analyses in this paper are available on GitHub (https://github.com/eabowman/Bowmanetal2023_BuffelGrass_FoliarFungalCommunities/settings) or https://zenodo.org/doi/10.5281/zenodo.11455748. Genetic data: Metabarcoding data are deposited in the SRA (BioProject PRJNA1010801). Benefits Generated: Benefits from this research accrue from the sharing of our data and results on public databases as described above.
