# Supplementary material for: Invasive Buffelgrass, Cenchrus ciliaris, Balances Opportunistic Acquisition of Foliar fungi With Host and Environmental Filtering in Its Introduced Range
Source: Mol Ecol. 2024 Dec 12;34(2):e17609. doi: 10.1111/mec.17609 (PMC11701881; doi:10.1111/mec.17609)
Supplement: Supplementary file 1 — Appendix S1. [file MEC-34-e17609-s001.pdf]

SUPPLEMENTAL INFORMATION

Invasive buffelgrass, *Cenchrus ciliaris*, balances opportunistic acquisition of foliar fungi with host and environmental filtering in its introduced range.

Running title: Foliar fungi and an invasive grass

Elizabeth A. Bowman, Nathan Jones, Christine V. Hawkes, Robert M. Plowes, Dino J. Martin, and Lawrence E. Gilbert

Table of Contents

Item	Pages
Supplementary Figure S1	2
Supplementary Figure S2	3
Supplementary Figure S3	4
Supplementary Table S1	5
Supplementary Table S2	6
Supplementary Table S3	7
Supplementary Table S4	8-10
Supplementary Table S5	11
Supplementary Results – for OTUs defined at 100% sequence similarity	12-20

Supplementary Figure S1: Correlation of climate variables to each other. Climate data was downloaded from WorldClim (Fick & Hijmans, 2017).

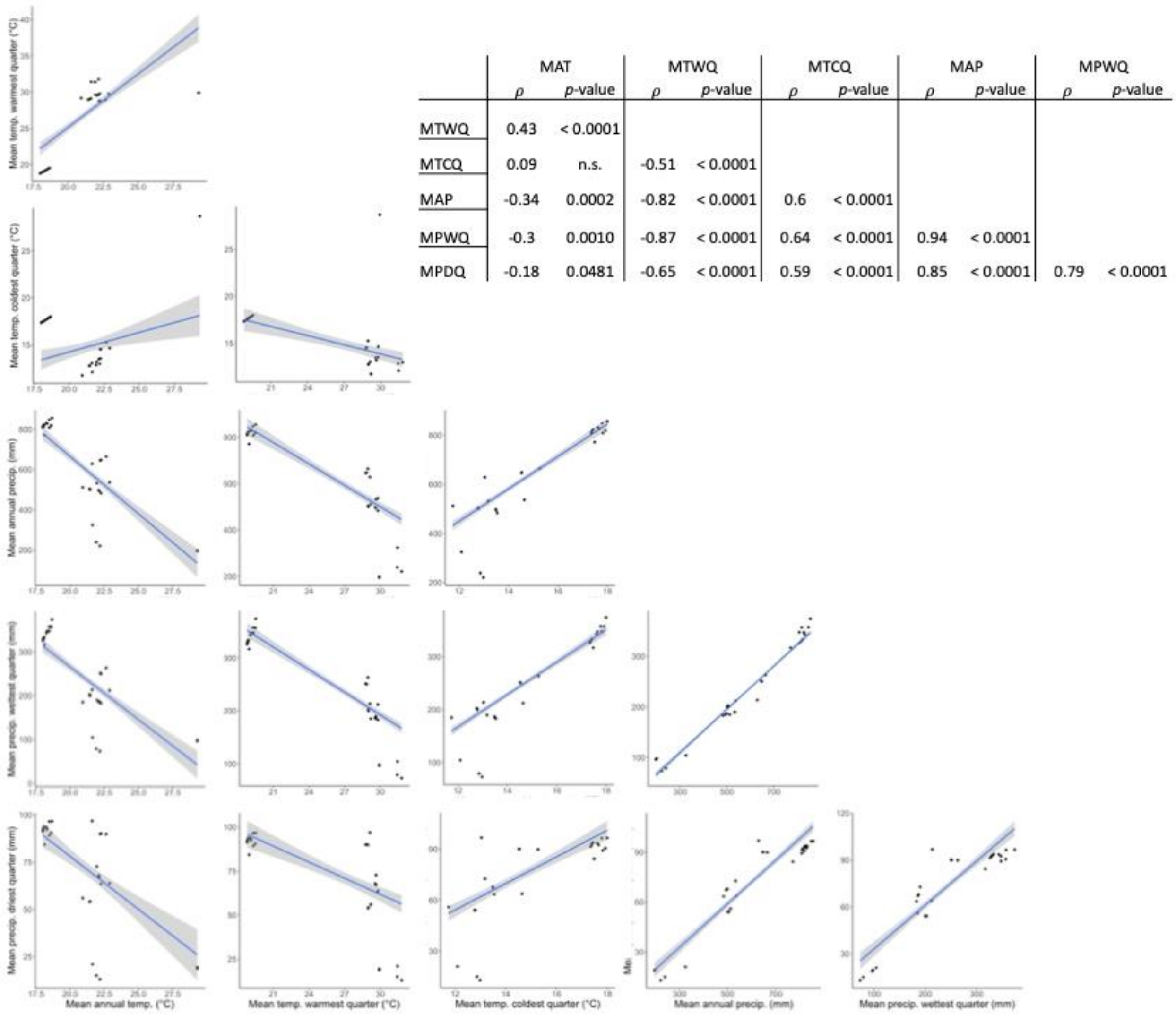

	MAT		MTWQ		MTCQ		MAP		MPWQ	
	ρ	*p*-value	*ρ*	*p*-value	*ρ*	*p*-value	*ρ*	*p*-value	*ρ*	*p*-value
MTWQ	0.43	< 0.0001								
MTCQ	0.09	n.s.	-0.51	< 0.0001						
MAP	-0.34	0.0002	-0.82	< 0.0001	0.6	< 0.0001				
MPWQ	-0.3	0.0010	-0.87	< 0.0001	0.64	< 0.0001	0.94	< 0.0001		
MPDQ	-0.18	0.0481	-0.65	< 0.0001	0.59	< 0.0001	0.85	< 0.0001	0.79	< 0.0001

Supplementary Fig. S2: Purported trophic mode of fungal foliar OTU unique to the introduced and native range of *C. ciliaris*, as well as shared between the two ranges. Trophic mode was defined with the fun^{fun} database (Flores-Moreno et al., 2019).

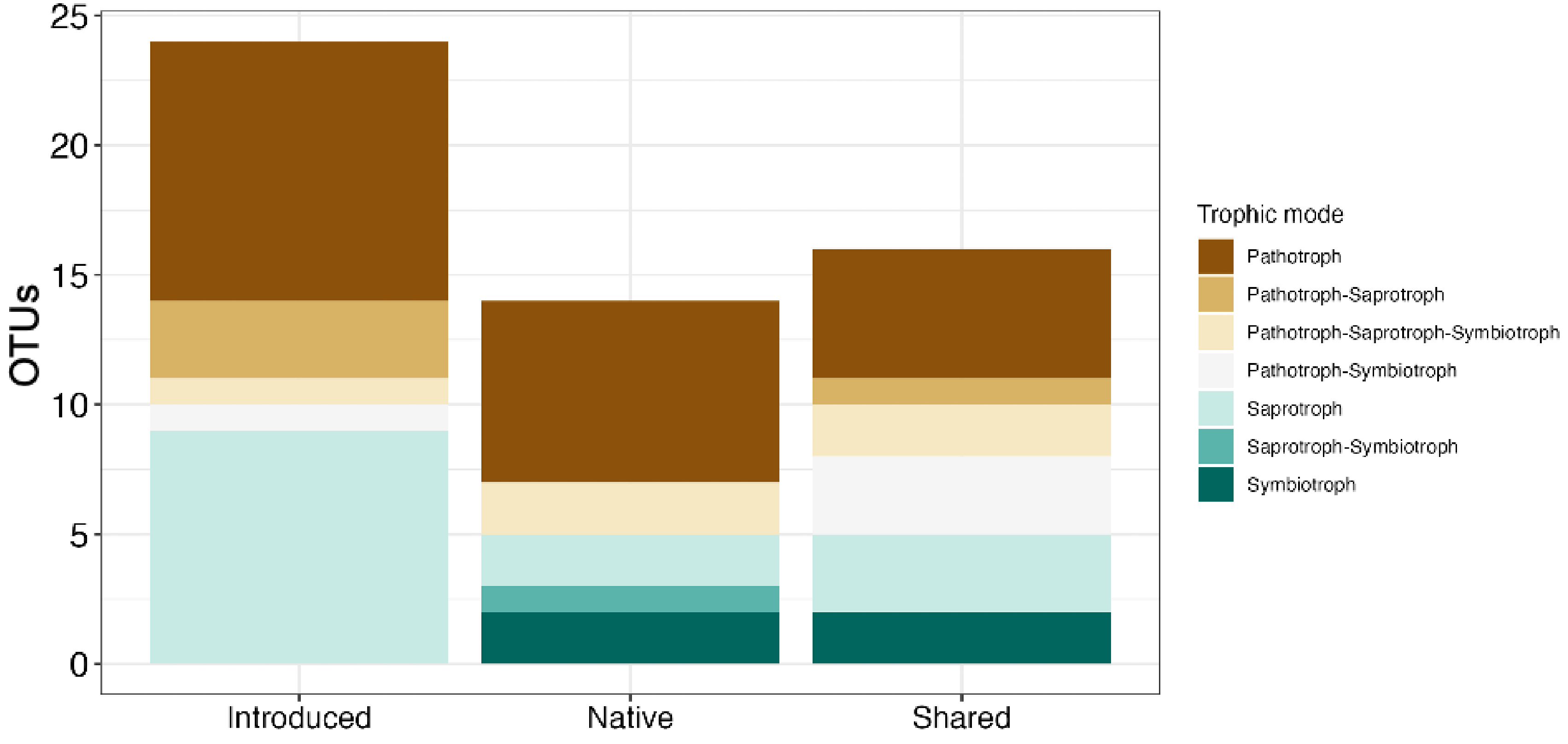

Supplementary Fig. S3: Occurrence of OTU shared between the native and introduced range of *C. ciliaris* on other native and non-native grasses within the introduced range. Grey = Non-native host; blue = native host.

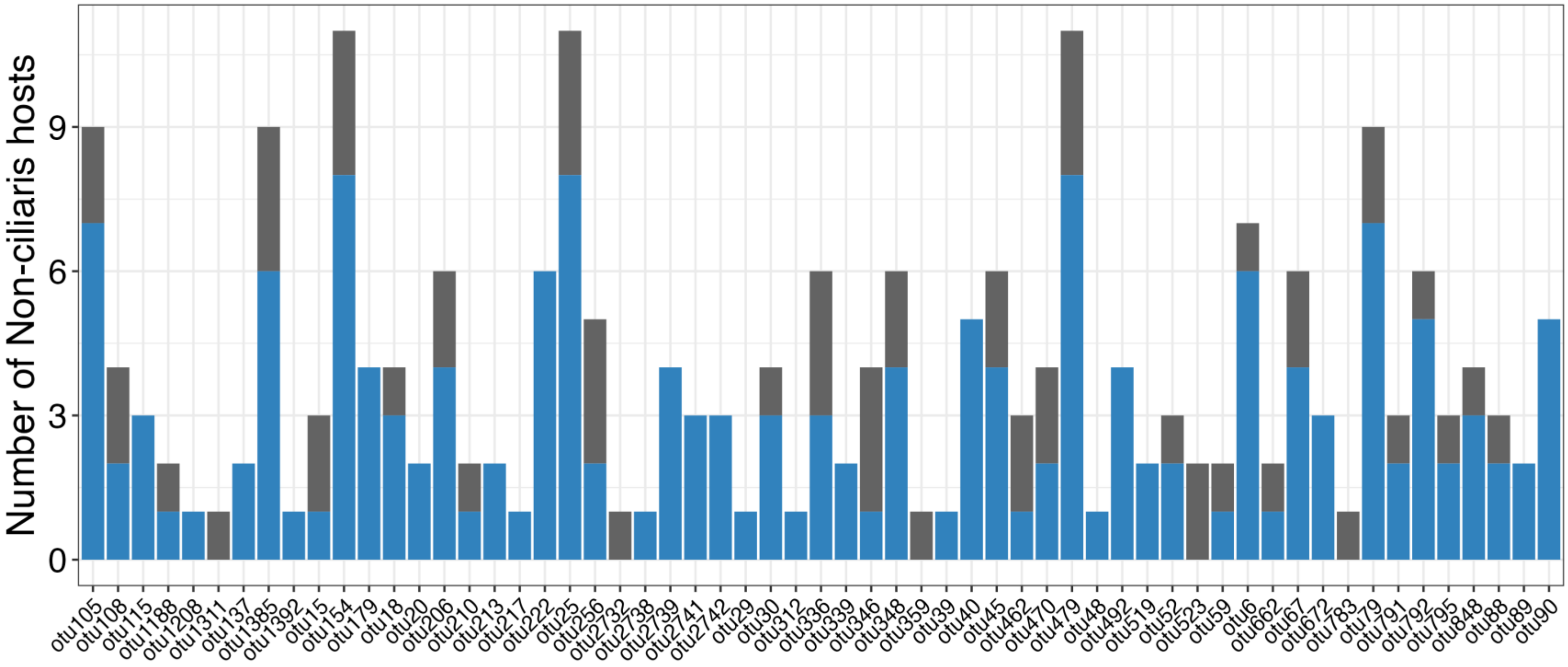

Supplementary Table S1: Locations and species of plants sampled. Numbers represent how many samples were collected at each location with totals for each plant species and location on the right and bottom of the table respectively. * = Non-native, invasive within Texas and Arizona, USA; ** = Non-native, non-invasive within Texas; all of the non-native species are native to Africa.

Site	Indio Faith	La Paloma	Retama	Moody Ranch	Raymondville	Daughtry	Falcon Lake	Catarina	Phoenix	Mpala	Turkana	
State	Texas								Arizona			
Country	U.S.A.									Kenya		
*Cenchrus ciliaris***	27	12	5	2	3	3	3	2	3	23	4	87
*Bothrioclhoa ischaemum***	-	2	-	-	-	1	-	-	-	-	-	3
Sporobolus sp.	-	-	2	1	-	-	-	-	-	-	-	3
Pappophorum bicolor	1	-	-	2	-	-	-	-	-	-	-	3
Setaria sp.	-	2	-	-	-	-	-	-	-	-	-	2
Hilaria mutica	2	-	-	-	-	-	-	-	-	-	-	2
Bouteloua curtipendula	2	-	-	-	-	-	-	-	-	-	-	2
Chloris sp.	-	-	-	1	1	-	-	-	-	-	-	2
*Heteropogon contortus**	-	1	-	-	-	-	-	-	-	-	-	1
*Panicum antidotale**	-	-	-	-	1	-	-	-	-	-	-	1
Aristida sp.	-	-	-	-	-	-	1	-	-	-	-	1
Sporobolus cryptandrus	-	1	-	-	-	-	-	-	-	-	-	1
	32	18	7	6	5	4	4	2	3	23	4	

Supplementary Table S2: Taxonomy of species sampled in the introduced range of *Cenchrus ciliaris* and accession numbers for *matK* sequences used to calculate host phylogenetic difference.

Species	Family	Subfamily	Supertribe	Tribe	Subtribe	NCBI accession
Cenchrus ciliaris	Poaceae	Panicoideae	Panicodae	Paniceae	Cenchrinae	FR821328
Setaria adhaerens	Poaceae	Panicoideae	Panicodae	Paniceae	Cenchrinae	FN908071
Megathyrsus maximum	Poaceae	Panicoideae	Panicodae	Paniceae	Panicinae	HE574152
Panicum sumatrense	Poaceae	Panicoideae	Panicodae	Paniceae	Panicinae	HE577885
Pappophorum vaginatum	Poaceae	Chloridoideae	-	Cynodonteae	Pappophorinae	JN681624
Hilaria cenchroides	Poaceae	Chloridoideae	-	Cynodonteae	Hilariinae	JN205313
Chloris barbata	Poaceae	Chloridoideae	-	Cynodonteae	Eleusininae	HE573963
Sporobolus airoides	Poaceae	Chloridoideae	-	Zoysieae	Sporobolinae	HE573973
Bouteloua curtipendula	Poaceae	Chloridoideae	Bouteloudinae	-	Boutelouinae	AF144578
Bothriochloa ischaemum	Poaceae	Pooideae	Andropogonodae	Andropogoneae	Andropogoninae	HE574006
Aristida adscensionis	Poaceae	Aristidoideae	-	Aristideae	-	AF164412

Supplementary Table S3: Trophic mode of OTUs associated with *C. ciliaris* within its introduced and native range as well as OTUs shared between ranges. Trophic mode was classified at the genus level. Percentage represents the proportion of OTUs representing that trophic mode within each range category. Trophic mode was defined with the fun^{fun} database (Flores-Moreno et al., 2019).

Range	Trophic mode	OTU count	Percentage of each range
Introduced	Pathotroph	10	9.3%
Native	Pathotroph	7	13.5%
Shared	Pathotroph	5	6.6%
Introduced	Pathotroph-Saprotroph	3	2.8%
Shared	Pathotroph-Saprotroph	1	1.3%
Introduced	Pathotroph-Saprotroph-Symbiotroph	1	0.9%
Native	Pathotroph-Saprotroph-Symbiotroph	2	3.8%
Shared	Pathotroph-Saprotroph-Symbiotroph	2	2.6%
Introduced	Pathotroph-Symbiotroph	1	0.9%
Shared	Pathotroph-Symbiotroph	3	3.9%
Introduced	Saprotroph	9	8.3%
Native	Saprotroph	2	3.8%
Shared	Saprotroph	3	3.9%
Native	Saprotroph-Symbiotroph	1	1.9%
Native	Symbiotroph	2	3.8%
Shared	Symbiotroph	2	2.6%
Introduced	Unclassified	84	77.8%
Native	Unclassified	38	73.1%
Shared	Unclassified	60	78.9%

Supplementary Table S4: Co-occurrence analysis of communities of foliar fungi associated with *C. ciliaris* in the introduced range. The analysis was run on a random subset of 27 samples. We repeated the analysis 100 times.

Repetition	Total pairs	Positive associations	% Positive	Negative associations	% Negative
1	1208	80	6.6	11	0.9
2	919	79	8.6	7	0.8
3	1629	139	8.5	7	0.4
4	1388	106	7.6	10	0.7
5	1541	87	5.6	18	1.2
6	1106	53	4.8	3	0.3
7	1057	63	6.0	12	1.1
8	1547	73	4.7	13	0.8
9	1370	86	6.3	12	0.9
10	1222	75	6.1	6	0.5
11	1194	81	6.8	12	1.0
12	1611	102	6.3	10	0.6
13	1120	67	6.0	4	0.4
14	1616	95	5.9	4	0.2
15	1318	107	8.1	5	0.4
16	1470	100	6.8	7	0.5
17	991	41	4.1	3	0.3
18	1049	81	7.7	4	0.4
19	1028	59	5.7	9	0.9
20	1345	93	6.9	16	1.2
21	1312	88	6.7	9	0.7
22	1270	54	4.3	11	0.9
23	1523	104	6.8	18	1.2
24	1370	88	6.4	13	0.9
25	1398	104	7.4	10	0.7
26	1221	92	7.5	9	0.7
27	1171	88	7.5	10	0.9
28	1264	86	6.8	6	0.5
29	1405	76	5.4	11	0.8
30	1271	77	6.1	6	0.5
31	1403	98	7.0	17	1.2
32	1435	95	6.6	15	1.0
33	1251	78	6.2	13	1.0
34	1205	60	5.0	21	1.7

35	1171	76	6.5	4	0.3
36	1392	72	5.2	6	0.4
37	1717	130	7.6	15	0.9
38	1376	59	4.3	11	0.8
39	1169	96	8.2	10	0.9
40	1165	68	5.8	2	0.2
41	1329	90	6.8	13	1.0
42	1364	97	7.1	15	1.1
43	1206	69	5.7	12	1.0
44	1059	45	4.2	11	1.0
45	1052	62	5.9	10	1.0
46	1588	92	5.8	15	0.9
47	1317	69	5.2	5	0.4
48	1158	100	8.6	13	1.1
49	1386	93	6.7	10	0.7
50	1278	95	7.4	12	0.9
51	1328	67	5.0	15	1.1
52	1406	109	7.8	11	0.8
53	1347	71	5.3	12	0.9
54	1540	97	6.3	12	0.8
55	1512	108	7.1	21	1.4
56	975	53	5.4	5	0.5
57	1365	73	5.3	12	0.9
58	1256	95	7.6	11	0.9
59	1335	75	5.6	12	0.9
60	912	50	5.5	10	1.1
61	1166	84	7.2	22	1.9
62	1181	81	6.9	6	0.5
63	1458	79	5.4	21	1.4
64	894	51	5.7	5	0.6
65	1542	92	6.0	16	1.0
66	1558	114	7.3	24	1.5
67	1231	91	7.4	6	0.5
68	938	69	7.4	14	1.5
69	1026	58	5.7	7	0.7
70	1331	88	6.6	18	1.4
71	1290	83	6.4	8	0.6
72	1119	50	4.5	6	0.5
73	1012	44	4.3	8	0.8
74	1005	60	6.0	7	0.7

75	1531	128	8.4	16	1.0
76	1607	102	6.3	15	0.9
77	1111	73	6.6	3	0.3
78	779	34	4.4	8	1.0
79	1117	76	6.8	10	0.9
80	1424	91	6.4	17	1.2
81	1453	101	7.0	3	0.2
82	1446	105	7.3	13	0.9
83	1157	85	7.3	6	0.5
84	961	36	3.7	4	0.4
85	1100	59	5.4	15	1.4
86	1494	104	7.0	11	0.7
87	1425	99	6.9	12	0.8
88	1288	74	5.7	13	1.0
89	1143	89	7.8	7	0.6
90	1210	81	6.7	6	0.5
91	1247	57	4.6	10	0.8
92	1299	101	7.8	7	0.5
93	1027	58	5.6	5	0.5
94	1516	106	7.0	26	1.7
95	1053	54	5.1	4	0.4
96	1073	75	7.0	5	0.5
97	1233	84	6.8	9	0.7
98	1108	68	6.1	6	0.5
99	1185	66	5.6	6	0.5
100	1533	136	8.9	10	0.7

Supplementary Table 5: Taxonomic classification and read abundance of OTUs that were unique to *C. ciliaris* and were not found in both native and non-native grasses sampled in the introduced range. Trophic mode was defined with the fun^{fun} database (Flores-Moreno et al., 2019).

Otu	Class	Order	Family	Genus	Trophic mode	Read abundance
Otu101	Sordariomycetes	Atractosporales	Atractosporaceae	Rubellisphaeria	Unclassified	1
Otu103	Eurotiomycetes	Chaetothyriales	Chaetothyriaceae	Ceramothyrium	Unclassified	1
Otu1094	Sordariomycetes	Hypocreales	Clavicipitaceae	Claviceps	Pathotroph	1
Otu1324	Saccharomycetes	Saccharomycetales	Debaryomycetaceae	Meyerozyma	Unclassified	1
Otu21	Dothideomycetes	Pleosporales	Lentitheciaceae	Darksidea	Unclassified	8
Otu2679	Lecanoromycetes	Teloschistales	Teloschistaceae	Rusavskia	Unclassified	2
Otu2709	Dothideomycetes	Capnodiales	unclassified	Elasticomyces	Unclassified	1
Otu271	Dothideomycetes	Pleosporales	Phaeosphaeriaceae	Septoriella	Unclassified	1
Otu2711	Sordariomycetes	Xylariales	Microdochiaceae	Microdochium	Unclassified	5
Otu2740	Dothideomycetes	Capnodiales	Neodevriesiaceae	Neodevriesia	Unclassified	1
Otu2761	Neolectomycetes	Neolectales	Neolectaceae	Neolecta	Saprotroph	1
Otu2777	Sordariomycetes	Hypocreales	Cordycipitaceae	Simplicillium	Unclassified	1
Otu371	Dothideomycetes	Botryosphaeriales	Botryosphaeriaceae	Sphaeropsis	Unclassified	1
Otu471	Dothideomycetes	Jahnulales	Aliquandostipitaceae	Jahnula	Unclassified	7
Otu665	Dothideomycetes	Capnodiales	Neodevriesiaceae	Neodevriesia	Unclassified	2
Otu731	Neolectomycetes	Neolectales	Neolectaceae	Neolecta	Saprotroph	1
Otu92	Dothideomycetes	Trypetheliales	Trypetheliaceae	Trypethelium	Unclassified	2

Supplementary Results (All figures and tables referenced in the Supplementary Results can be found on pages 14-20.)

Based on defining OTUs at 100% sequence similarity, we documented 400 putative species of fungi associated with leaf tissues of *Cenchris ciliaris* (135 species with greater than 10 occurrences). We found 74 OTUs shared between *C. ciliaris*'s native range in Kenya and its introduced range in the United States. There were 163 OTUs unique to Kenya and 157 unique to the United States. Overall, there was no significant difference in OTU richness although the introduced range had slightly higher OTU richness (mean 22.8 ± 9.4) than the native range (mean 19.0 ± 8.5). Within the native range, there was a difference OTU richness with Mpala having slightly higher OTU richness than Turkana (Fig. 1; Table 1). Moody Ranch, which had the highest OTU richness in the introduced range, had eight times more putative species than the site with the lowest OTU richness, Phoenix, AZ (Fig. 1; Table 1).

Overall, range explained 60.1% of the variation in fungal communities associated with *C. ciliaris* ($p = 0.001$). Consistent with this, foliar fungal communities were more similar to each other within each range (native range: 0.85 ± 0.1; introduced range: 0.85 ± 0.1) than between the native and introduced range (0.91 ± 0.1) (Fig. 2). Range, geographic distance, and climate (represented by MPWQ) explained 16.6% of variation in the foliar fungal communities. Range explained most of the variation (7.5%) followed by MPWQ, range * MPWQ, and range * geographic distance (Table 2).

Within the introduced range, MAP explained most of the variation within the foliar fungal community (6.8%) followed by MAP * geographic distance (Fig. 3, Table 3). Total variation

explained by our model was 11.0%. When native status of the plant species was included, it was insignificant. When foliar fungal communities were compared between host species and within a single host species, communities within the same host were more similar than between heterospecific host species (Fig. 4). Foliar fungal communities in *C. ciliaris* were more dispersed at the species level (within group: 0.83 ± 0.17 Jaccard dissimilarity) than communities found in other species (*B. ischaemum*, within: 0.58 ± 0.44; *P. bicolor*, within: 0.40 ± 0.31; *Sporobolus* sp., within: 0.56 ± 0.42; Fig. 4).

Figure S1: OTU richness was significantly different between sampled sites in the native (grey) and introduced range (blue). ANOVA: $F_{10,61} = 6.47$, $p < 0.0001$)

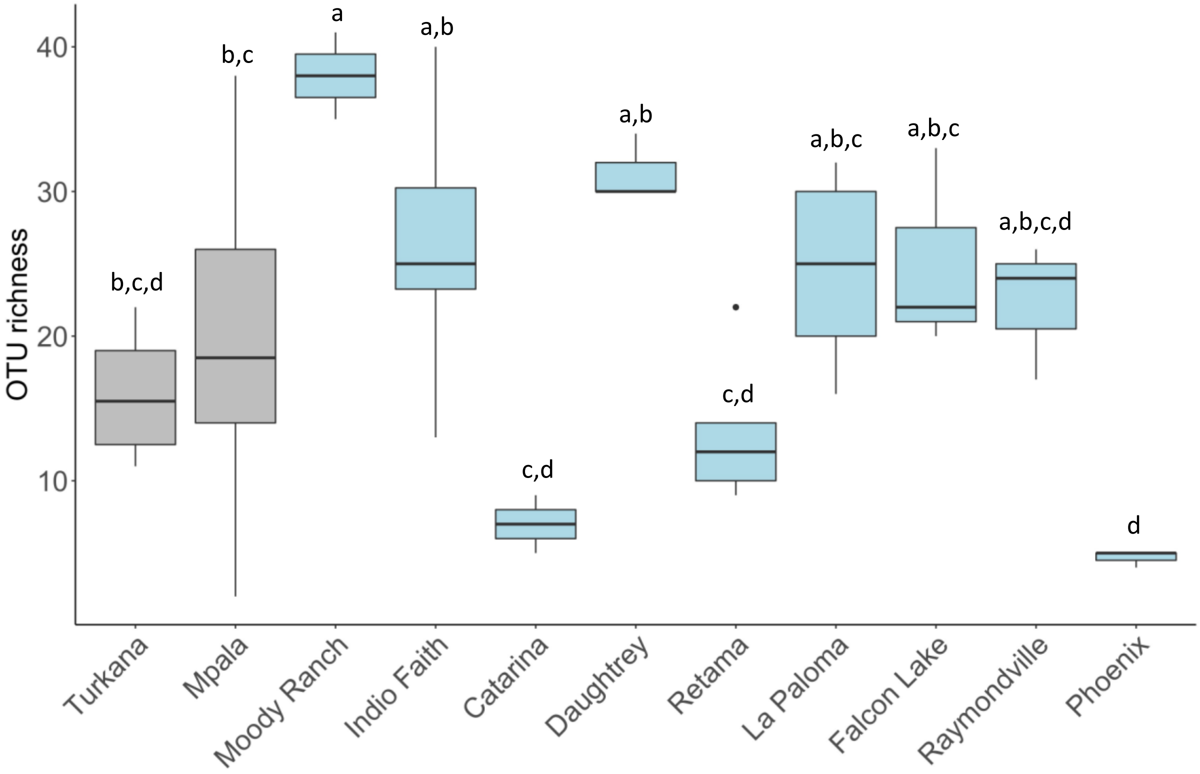

Figure S2: Comparison of foliar fungal communities associated with *Cenchrus ciliaris* in its native range, Kenya, and its introduced range, Texas and Arizona. Panel A is a pairwise comparison of fungal communities associated with *C. ciliaris* across the native and introduced range (Overall) and within each range (Introduced and Native range; Wilcoxon test: W = 1559308, $p < 0.0001$). Panel B is an NMDS ordination showing community similarity across both ranges (stress = 0.1716). Circles = introduced range; triangles = native range; blue represents the introduced range (light blue = Arizona, dark blue = Texas); grey represents the native range (light grey = Mpala, dark grey = Turkana).

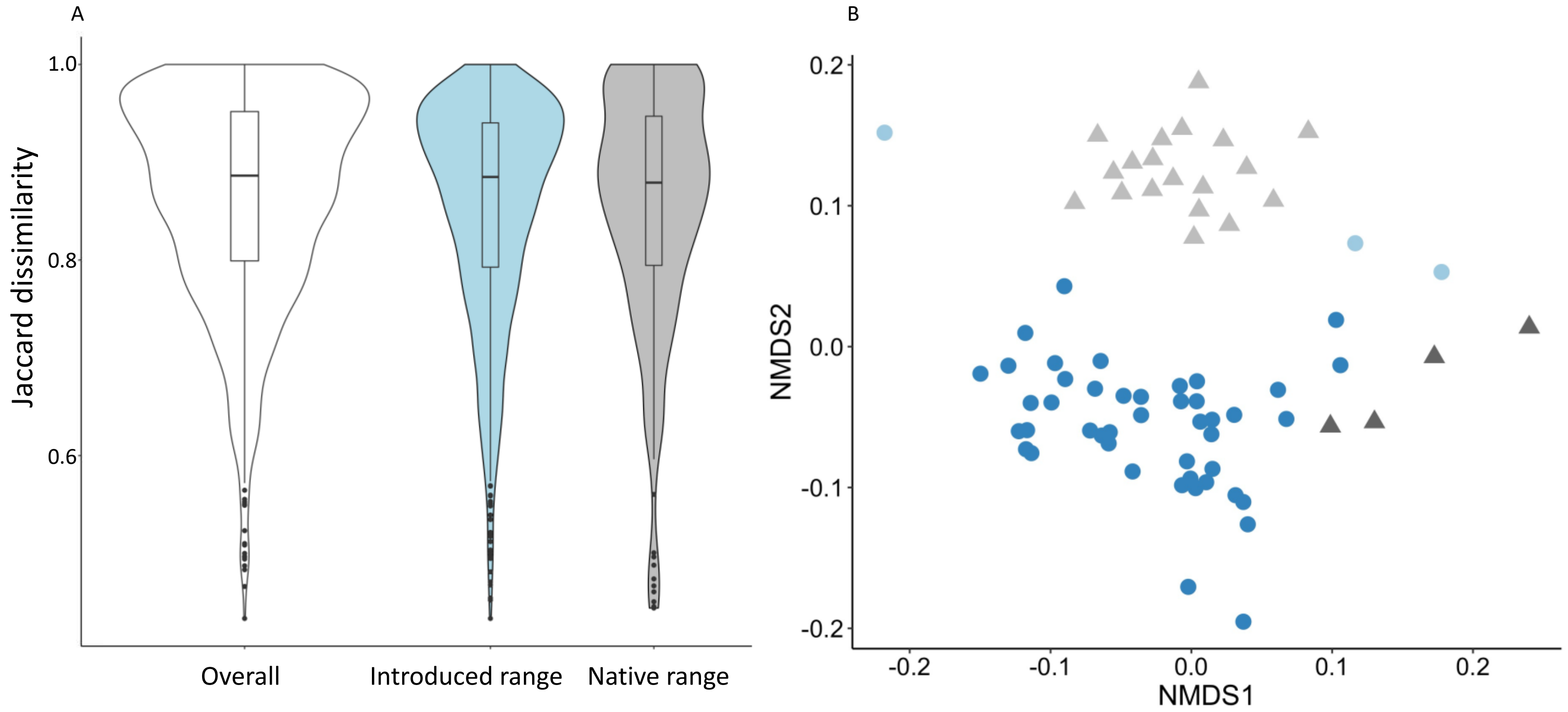

Figure S3: NMDS ordinations of foliar fungal communities associated with grass species sampled across Texas (stress 0.1736). Panel A shows points colored based on mean annual precipitation (MAP). Panel B shows points colored by host species represented in the phylogeny in panel C. Numbers at branch points are bootstrap support values.

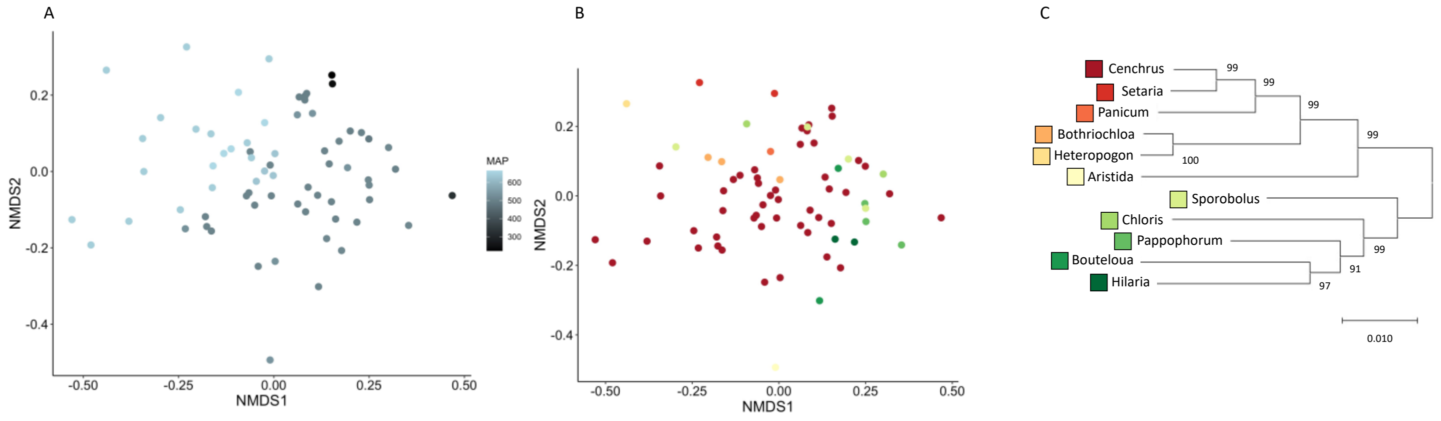

Figure S4: Pairwise comparison of Jaccard similarity of *C. ciliaris*, *B. ischaemum*, *P. bicolor*, *Sporobolus* sp. in Texas. Between represents comparisons between these four species; within represents within each individual species. *B. ischaemum* and *C. ciliaris* are non-native plants.

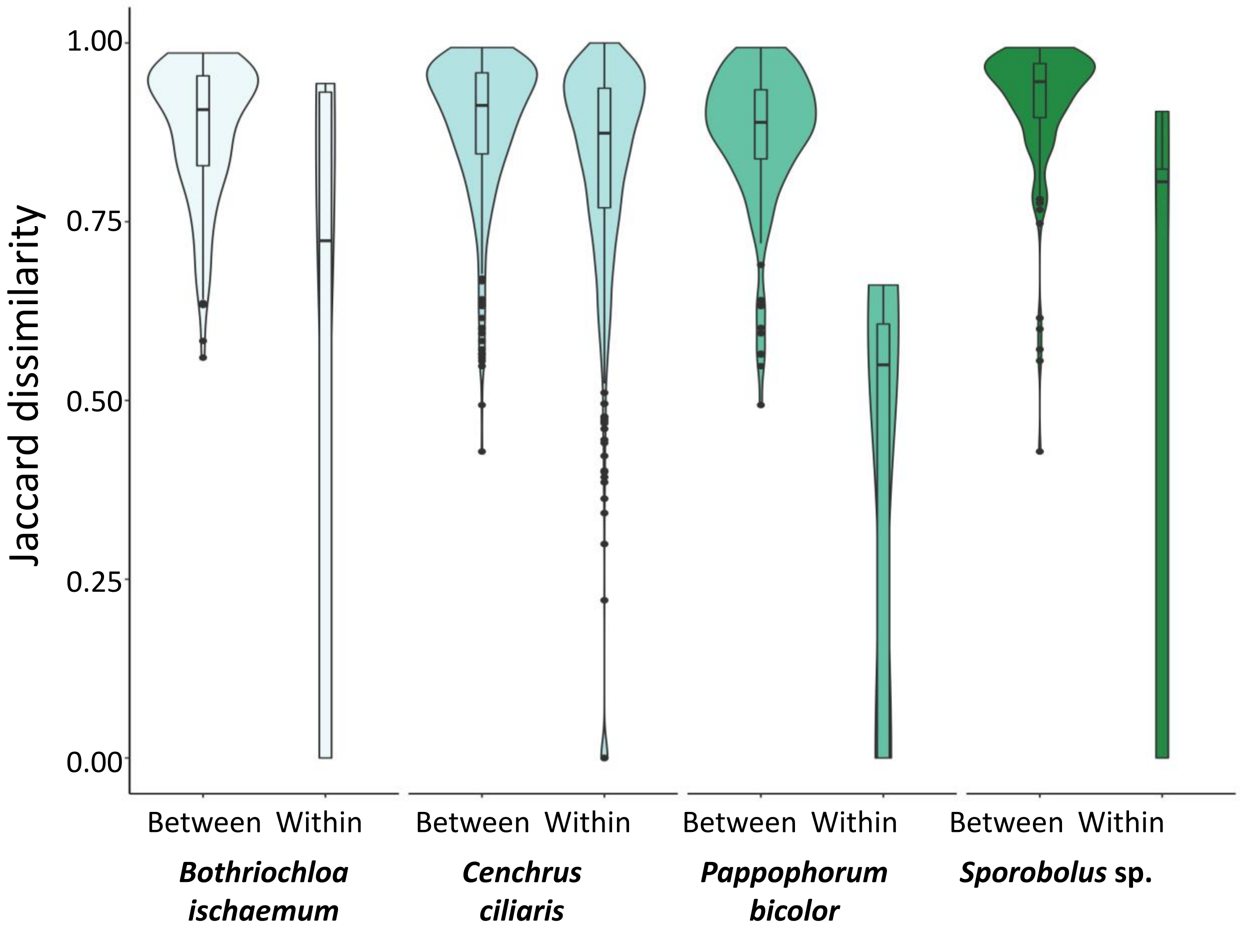

Table S1: OTU richness of foliar fungi associated with *Cenchrus ciliaris* by site. Mean and one standard deviation.***values need to be changed for the 100% sequence similarity dataset

Range	State	Site	OTU richness
Invasive	TX	Indio Faith	25.9 ± 6.7
	TX	La Paloma	24.6 ± 5.6
	TX	Daughtrey	31.3 ± 2.3
	TX	Falcon Lake	25.0 ± 7.0
	TX	Raymondville	22.3 ± 4.7
	TX	Retama	13.4 ± 5.2
	TX	Moody Ranch	38.0 ± 4.2
	TX	Catarina	7.0 ± 2.8
	AZ	Phoenix	4.7 ± 0.6
Native	Kenya	Mpala	19.7 ± 9.0
	Kenya	Turkana	16.0 ± 5.0

Table S2: PERMANOVA analysis of variation in the foliar fungal community associated with *Cenchrus ciliaris* in its introduced range and native range. The model used here was range * geographical distance (geo. dist.) * mean precipitation of the wettest quarter (MPWQ).

	F	*P*	R^2
Range	6.14	0.0020	0.075
MPWQ	2.93	0.0030	0.036
Range * MPWQ	2.93	0.0010	0.036
Geo. dist. * MPWQ	1.80	N.S.	0.022
Geo. dist.	1.75	N.S.	0.021
Range * Geo. dist.	1.62	0.0510	0.020
Range * Geo. dist. * MPWQ	0.93	N.S.	0.011
Total variation explained			0.1659

Table S3: PERMANOVA analysis of variation in the foliar fungal community associated with plant species in Texas. The model used here was host phylogeny * mean annual precipitation (MAP) * geographical distance (geo. dist.). See Supplementary Table S3 for analysis conducted with native status of plants included (i.e. native versus non-native to Texas).

	F	*P*	R^2
MAP	5.15	0.0230	0.068
Geo. dist.	2.84	0.2000	0.037
MAP * Geo. dist.	1.79	0.0450	0.024
Host phylogeny	1.77	0.1410	0.023
Host phylogeny * MAP * Geo. dist.	1.44	0.0220	0.019
Host phylogeny * Geo. dist.	1.08	0.8340	0.014
Host phylogeny * MAP	0.99	0.6210	0.013
Total variation explained			0.110
